# Early intubation and clinical outcomes in patients with severe COVID-19: a systematic review and meta-analysis

**DOI:** 10.1186/s40001-022-00841-6

**Published:** 2022-11-03

**Authors:** Hyeon-Jeong Lee, Joohae Kim, Miyoung Choi, Won-Il Choi, Joonsung Joh, Jungeun Park, Junghyun Kim

**Affiliations:** 1 Division of Healthcare Technology Assessment Research, National Evidence-Based Healthcare Collaborating Agency, Seoul, Republic of Korea; 2 Division of Pulmonary and Critical Care Medicine, Department of Internal Medicine, National Medical Center, Seoul, Republic of Korea; 3 Department of Internal Medicine, Myongji Hospital, Hanyang University, Gyeonggi-do, Republic of Korea; 4 Present Address: Division of Pulmonary, Allergy and Critical Care Medicine, Department of Internal Medicine, Hallym University Dongtan Sacred Heart Hospital, Hallym University College of Medicine, Hwaseong, Republic of Korea

**Keywords:** Early intubation, COVID-19, Meta-analysis, Mechanical ventilation

## Abstract

**Background:**

Evidence regarding the timing of the application of mechanical ventilation among patients with severe coronavirus disease (COVID-19) is insufficient. This systematic review and meta-analysis aimed to evaluate the effectiveness of early intubation compared to late intubation in patients with severe and critical COVID-19.

**Methods:**

For this study, we searched the MEDLINE, EMBASE, and Cochrane databases as well as one Korean domestic database on July 15, 2021. We updated the search monthly from September 10, 2021 to February 10, 2022. Studies that compared early intubation with late intubation in patients with severe COVID-19 were eligible for inclusion. Relative risk (RR) and mean difference (MD) were calculated as measures of effect using the random-effects model for the pooled estimates of in-hospital mortality, intensive care unit (ICU) length of stay (LOS), duration of mechanical ventilation (MV), hospital LOS, ICU-free days, and ventilator-free days. Subgroup analysis was performed based on the definition of early intubation and the index time. To assess the risk of bias in the included studies, we used the Risk of Bias Assessment tool for Non-randomized studies 2.0.

**Results:**

Of the 1523 records identified, 12 cohort studies, involving 2843 patients with severe COVID-19 were eligible. There were no differences in in-hospital mortality (8 studies, *n* = 795; RR 0.91, 95% CI 0.75–1.10, *P* = 0.32, *I*^2^ = 33%), LOS in the ICU (9 studies, *n* = 978; MD −1.77 days, 95% CI −4.61 to 1.07 days, *P* = 0.22, *I*^2^ = 78%), MV duration (9 studies, *n* = 1,066; MD −0.03 day, 95% CI −1.79 to 1.72 days, *P* = 0.97, *I*^2^ = 49%), ICU-free days (1 study, *n* = 32; 0 day vs. 0 day; *P* = 0.39), and ventilator-free days (4 studies, n = 344; MD 0.94 day, 95% CI −4.56 to 6.43 days, *P* = 0.74, *I*^2^ = 54%) between the early and late intubation groups. However, the early intubation group had significant advantage in terms of hospital LOS (6 studies, *n* = 738; MD −4.32 days, 95% CI −7.20 to −1.44 days, *P* = 0.003, *I*^2^ = 45%).

**Conclusion:**

This study showed no significant difference in both primary and secondary outcomes between the early intubation and late intubation groups.

*Trial registration* This study was registered in the Prospective Register of Systematic Reviews on 16 February, 2022 (registration number CRD42022311122).

**Supplementary Information:**

The online version contains supplementary material available at 10.1186/s40001-022-00841-6.

## Background

The treatment of severe pneumonia and acute respiratory distress syndrome (ARDS) following the coronavirus disease (COVID-19) pandemic is becoming a challenge [1]. Progressive respiratory failure develops in many patients with severe COVID-19, soon after the onset of dyspnea and hypoxemia. These patients commonly meet the ARDS criteria, defined as acute onset of bilateral infiltrates, severe hypoxemia, and lung edema that is not fully explained by cardiac failure or fluid overload [2, 3]. In severe and critical COVID-19, it is clinically difficult to determine the appropriate timing for invasive mechanical ventilation, resulting in the provision of different treatments based on by physicians’ own experiences and preferences.

Some expert recommendations based on previous studies have suggested early intubation for patients with severe and critical COVID-19, aimed to protect health personnel from cross-infection, reduce complications from tracheal intubation, and prevent self-induced lung injury (SILI) [4–7]. SILI generates an early phase of ARDS; the high transpulmonary pressures associated with spontaneous vigorous inspiratory effort may contribute to lung damage before the patient becomes fatigued or is sedated [8–10]. To prevent lung injury, it is recommended that SILI is prevented in the early stage of ARDS through various approaches such as supplemental oxygen, continuous positive airway pressure (CPAP), non-invasive ventilation (NIV), high-flow nasal cannula (HFNC), awake prone positioning, and target non-vigorous breathing. This is the rationale for the need for early tracheal intubation [9, 10].

Early intubation may pose a risk of generating viral aerosols and self-induced lung injury. Moreover, the background for early intubation is still based on theoretical physiology, and the clinical evidence of early intubation is still not fully considered [11–13]. Although several studies on the prognostic difference between early and late intubations have been reported [14–17], there are inevitable differences in the primary outcome, definition of early and late intubations, and the study designs, resulting in inconsistent results. Therefore, international guidelines, including those of the National Institute of Health, World Health Organization, and Australia, reveal no clear timeline for the recommendation on the application of mechanical ventilation (MV) among patients with severe COVID-19 [18–20]. Therefore, we aimed to explore the effect of the timing of MV on the clinical course and prognosis of patients with severe and critical COVID-19 through a systematic review and meta-analysis.

## Methods

This study was conducted according to the recommendations outlined in the Preferred Reporting Items for Systematic Reviews and Meta-Analyses 2020 guidelines [21] (Additional File 1). This study was registered in the Prospective Register of Systematic Reviews on 16 February, 2022 (registration number CRD42022311122).

### Eligibility criteria

The inclusion criteria were as follows: (1) population: studies targeting patients with severe COVID-19; (2) intervention and comparator: studies comparing early intubation to late intubation; (3) outcomes: studies reporting clinical outcomes (in-hospital mortality, length of stay, duration of MV, etc.); (4) studies published after 2020; (5) a randomized clinical trial or an observational study with a comparator group; and (6) full-text articles in English or Korean. The exclusion criteria were as follows: (1) studies that did not target patients with confirmed COVID-19, (2) studies that did not compare early and late intubation, (3) studies that did not report our outcomes of interest, and (4) duplicated studies.

### Information sources and search strategy

We searched the following electronic databases: international databases (Ovid MEDLINE, Ovid EMBASE, the Cochrane Central Register of Controlled Trials), and the Korean domestic database (KMBASE) on July 15, 2021. Since new evidence on early intubation of patients COVID-19 patients is continuously published, we performed the search monthly from September 10, 2021 to February 10, 2022 to update the included studies. We searched Ovid MEDLINE for updates and reference lists of previously published related reviews. We used both controlled terms and text words such as (2019-nCoV OR COVID-19 OR Wuhan) AND (intubation, intratracheal OR intubation, endotracheal OR early intubation OR early endotracheal intubation). The search strategy is presented in Additional File 2.

### Selection process

Two pairs of authors (HJL, JoK, WIC, and JJ) screened the title and abstract of retrieved citations using Covidence (https://www.covidence.org/). Each author independently assessed the eligibility of the identified studies, and conflicts resolved by discussion. Full texts were assessed by two authors (HJL and JoK) for the final decision on inclusion or exclusion. Any disagreement between the two authors was resolved through a discussion with a third author (MC).

### Data items and extraction

The following data were extracted from the eligible studies using an electronic spreadsheet (Microsoft Excel) of a pre-designed data extraction form: author, publication year, study design, study country, study setting, COVID-19 severity, number in each arm, timing of intubation, definition of early intubation, and outcomes of interest. One author (JP) extracted the data extraction, and another two authors (JJ and WIC) independently evaluated the data.

### Study outcomes

The primary outcome was all-cause mortality during hospitalization. The secondary outcomes were length of stay (LOS) in the intensive care unit (ICU), duration of MV, hospital LOS, ICU-free days, and ventilator-free days.

### Study risk of bias assessment

A validated tool was used to evaluate the risk of bias according to the study design. The Risk of Bias Assessment Tool for Non-randomized Studies (RoBANS) 2.0 [22], which is an update of RoBANS 1.0 [23] was used for nonrandomized studies. This tool contains eight domains, including the possibility of target group comparisons, target group selection, confounders, exposure measurement, blinding of assessors, outcome assessment, incomplete outcome data, and selective outcome reporting. Each domain was assessed as having low, high, or unclear risk of bias. Quality assessments of the studies were conducted by two authors (WIC and JJ) independently, and disagreements were resolved through a discussion with a third author (MC).

### Effect measures and synthesis methods

Based on the data extraction results, a meta-analysis was performed as follows. Relative risks (RR) with 95% confidence interval (CI) for discrete outcome data and mean differences (MD) with 95% CI for continuous outcome data were calculated using the random-effects model because of the heterogeneity across studies. Statistical significance was set at *p* < 0.05. To assess between-study heterogeneity, we constructed forest plots and calculated *I*^2^ statistics, with a value of > 75% considered as high heterogeneity [24]. A subgroup analysis was performed based on the definition of early intubation (intubated before 24 h or 48 h from the index time [ARDS onset or ICU admission]) and based on the index time for intubation in each study group. To synthesize the data, we used Review Manager 5.4 (RevMan, The Cochrane collaboration, Oxford, UK) software for the meta-analysis.

### Certainty of evidence assessment

We used the Grading of Recommendations, Assessment, Development, and Evaluation (GRADE) [25] to assess the certainty of evidence of in-hospital mortality, LOS in the ICU, MV duration, and hospital LOS. The GRADE includes five reasons for rating down the certainty in effect estimates (risk of bias, imprecision, inconsistency, indirectness, and publication bias) and three reasons for rating up certainty. Two authors assessed (WIC and JJ) the certainty of evidence as high, moderate, low, or very low, and discrepancies were resolved through discussions with a third author (MC).

## Results

### Study selection

**Fig. 1:**
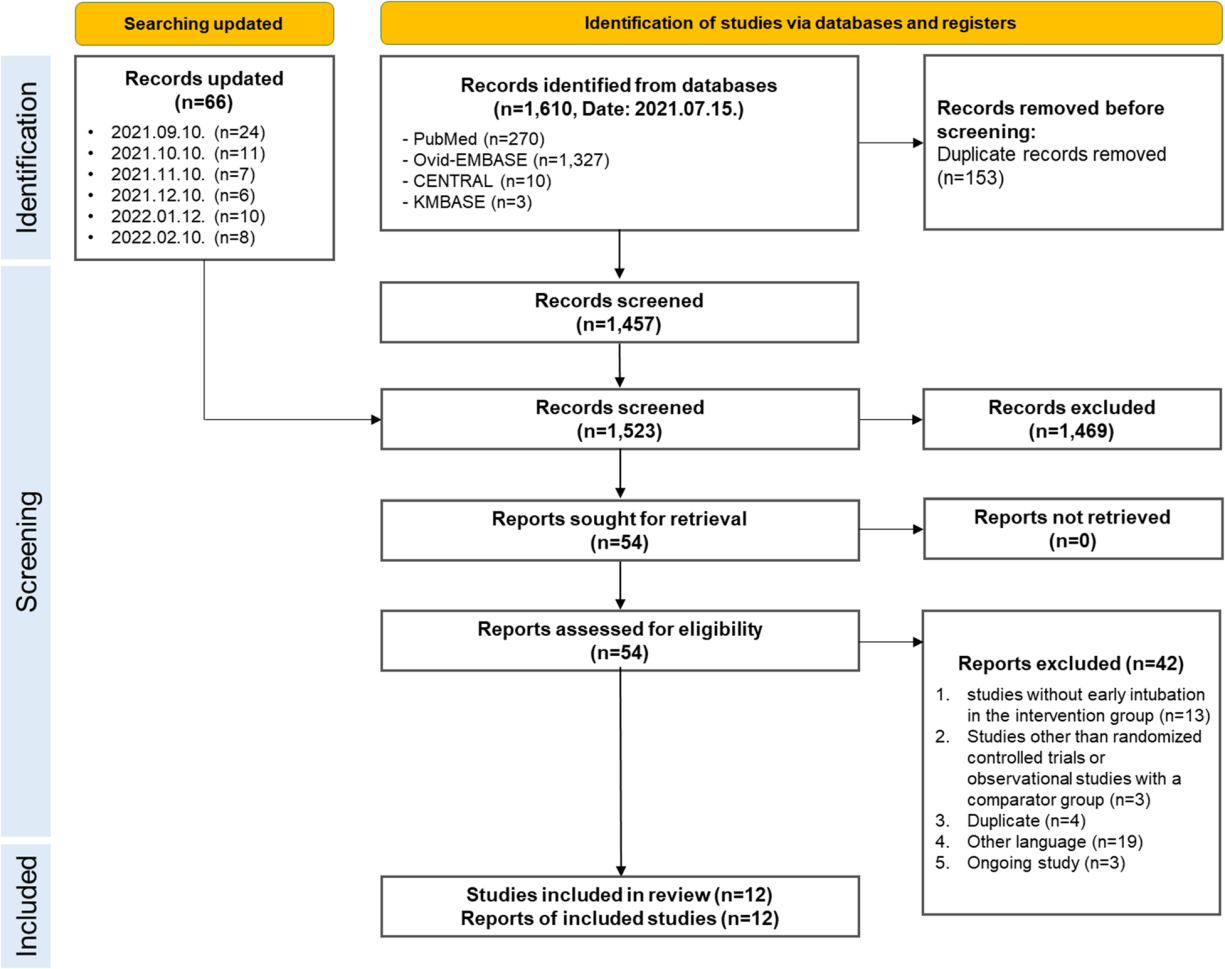
PRISMA flowchart

A total of 1610 records were identified in July 2021 through the search strategy, and 153 duplicate records were removed before screening. Sixty-six records were subsequently included through monthly searches until February, 2022, totaling 1523 studies. Of the 1523 records, 1,469 were excluded after screening the titles and abstracts. Subsequently, the full texts of 54 reports were reviewed. After reviewing the eligibility of the original texts, 12 cohort studies [14–17, 26–33] were included, and there were no randomized controlled trials (Fig. 1). The list of excluded studies and reasons for exclusion are presented (Additional file 3).

### Study characteristics

The characteristics of the included studies are summarized in Table 1. Four prospective cohort studies [16, 27, 31, 33] included 504 patients (sample size range, 32–205), and eight retrospective cohort studies [14, 15, 17, 26, 28–30, 32] included 2,339 patients (sample size range, 39–1618). Six studies [14, 15, 26, 28, 30, 32] were performed in the US, three [27, 31, 33] in Europe, two [17, 29] in Asia, and one [16] in Chile. Seven studies [14–17, 26, 30, 33] were single-center studies, while the others were multi-center studies. The index time for intubation and the definition of early intubation varied. The index time for intubation was defined according to the onset of acute respiratory failure in five studies [15, 26, 29, 30, 33] and ICU admission in three studies [17, 28, 31]. Five studies [14, 26, 28, 29, 31] defined early intubation as intubation within 24 h and five studies [16, 17, 27, 30, 32] as within 48 h from the index time.

**Table 1:** The basic characteristics of studies included in this review

First author, publication year	Study design/setting	Country	Patients Total (early intubation /late intubation) (N)	Age Early intubation/late intubation (yr, IQR or SD)	Male Early intubation/late intubation (%)	Pharmacological treatment Early intubation/late intubation (%)	Definition of early intubation	Index time
González, 2022 [27]	Prospective/multi-center	Spain	205 (140/65)	63.0 (54.0–71.0) /63.0 (59.0–69.0)	75.7/70.8	Steroids: 90.9/87.3 Remdesivir: 25/30	< 48 h	First respiratory support
Bavishi, 2021 [14]	Retrospective/single center	US	54 (30/24)	58 (42–69) /62 (50–69)	70/66	Steroids: 23.3/33.3 Anti-interleukin-6: 26.7 / 29 Hydroxychloroquine: 3.3 / 17 Remdesivir: 23.3/21	< 24 h	Hospital admission
Fayed, 2021 [26]	Retrospective/single center	US	110 (55/55)	64.3 (12.7)/63.5 (15.5)	NR	Steroids: 90.9/87.3 Remdesivir: 25/30	< 24 h	ARDS onset
Mellado-Artigas, 2021 [31]	Prospective/multi-center	Spain, Andorra	84 (61/23)	61 (11) /63 (9)	52/61	Immunosuppression: 3.3/6.6	< 24 h	ICU admission
Pandya, 2021 [15]	Retrospective/single center	US	75 (37/38)	65.92 (14.79) /64.05 (13.87)	48.84/51.16	NR	1.27 days	ARDS onset
Parish, 2021 [32]	Retrospective/multi-center	US	1628 (807/821)	> 65yrs 44.5%/44.7%	65.6/64.7	NR	< 48 h	Triage in the emergency department
Vera, 2021 [16]	Prospective/single center	Chile	183 (88/95)	59 (53–66) /64 (55–71)	71/74	Steroids: 34/41	< 48 h	Hospital admission
Zirpe, 2021 [17]	Retrospective/single center	India	147 (75/72)	58 (50–69) /59 (52–67)	74.6/73.6	NR	< 48 h	ICU admission
Hernandez-Romieu, 2021 [28]	Retrospective/multi-center	US	175 (133/42) -Intubated within 8 h (*n* = 76) -Intubated within 8 ~ 24 h (*n* = 57)	Intubated < 8 h 67 (56–76), 8–24 h 65 (55–73) /67 (57–77)	Intubated < 8 h 50, 8–24 h 59.6 /59.5	NR	< 24 h	ICU admission
Lee, 2020 [29]	Retrospective/multi-center	South Korea	39 (23/16)	72 (64–76) /66 (59–77)	60.9/62.5	Steroids: 78.3/93.8 Hydroxychloroquine: 87/87.5 Lopinavir–ritonavir: 87/68.8 Darunavir–cobicistat: 13/31.2	< 24 h	ARDS onset
Matta, 2020 [30]	Retrospective/single center	US	111 (76/35)	69.79 (12.15) /65.03 (8.37)	55/51	Steroids: 74/71 Tocilizumab: 30/31 Hydroxychloroquine: 65/77 Remdesivir: 15/14	< 48 h	ARDS onset
Siempos, 2020 [33]	Prospective/single center	Greece	32 (14/18)	63 (57–69) /64 (57–74)	57/92	NR	The remaining intubated patients except for the late intubation group (late intubation group: non-rebreather mask for ≥ 24 h or HFNC/NIV for any period of time in an attempt to avoid intubation)	ARDS onset

*IQR* interquartile range, *SD* standard definition, *NR* not reported, *ARDS* acute respiratory distress syndrome, *ICU* intensive care unit, *HFNC* high flow nasal cannula, *NIV* non-invasive ventilation

### Risk of bias in studies

**Fig. 2:**
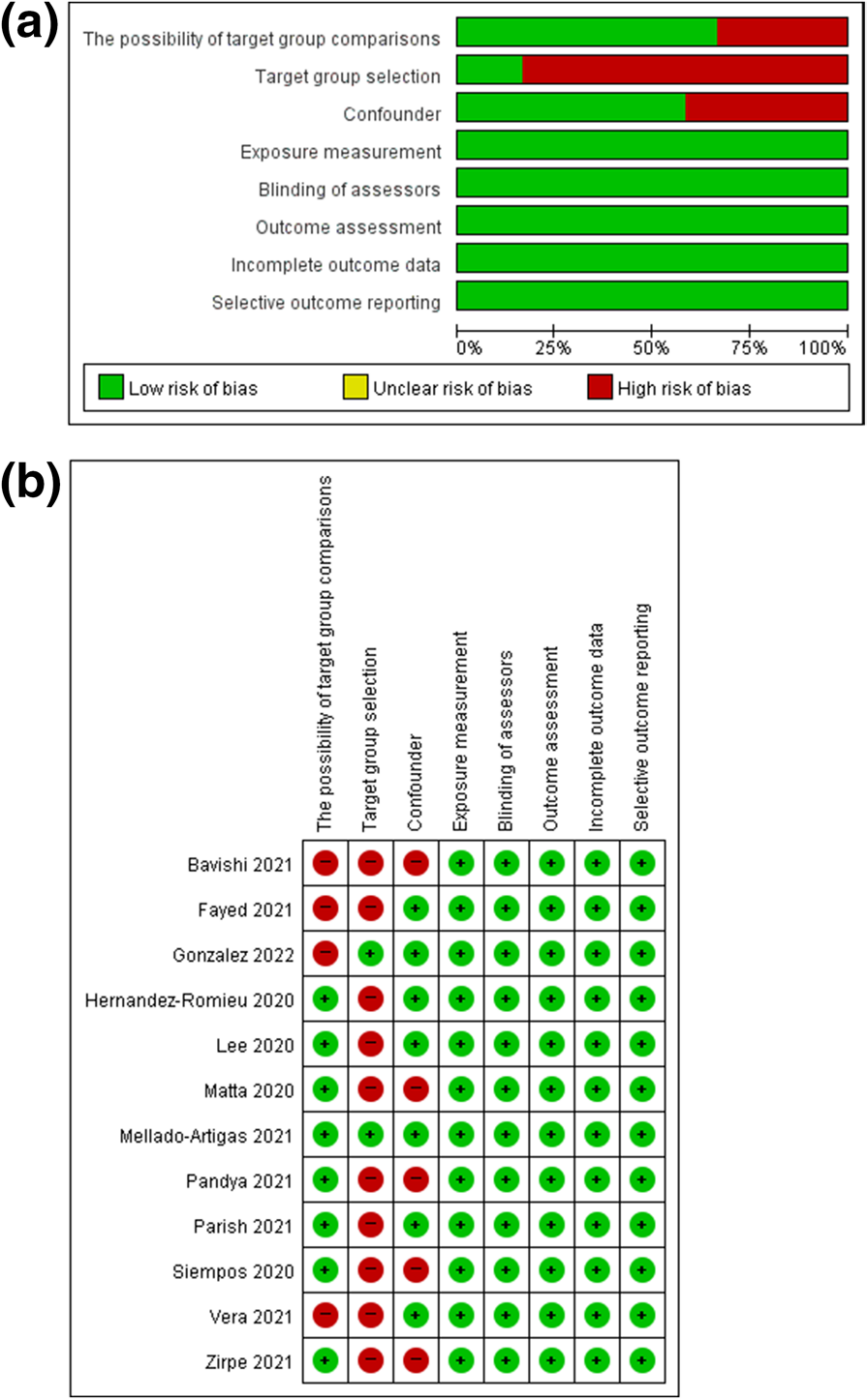
Risk of bias of included studies. **a** Risk of bias graph: review authors' judgements about each risk of bias item presented as percentages across all included studies. **b** Risk of bias summary: review authors' judgements about each risk of bias item for each included study

Four studies [14, 16, 26, 27] were rated as having a high risk of bias in the domain of possibility of target group comparisons, ten studies [14–17, 26, 28–30, 32, 33] as high risk of bias in the domain of target group selection, and five studies [14, 15, 17, 30, 33] as high risk of bias in the domain of confounders (Fig. 2). Although there were some concerns in the domain of target group selection, serious problems did not occur because the domains of exposure measurement, blinding of assessors, and outcome assessment were assessed as having a low risk of bias.

### Primary outcome

#### In-hospital mortality

**Fig. 3:**
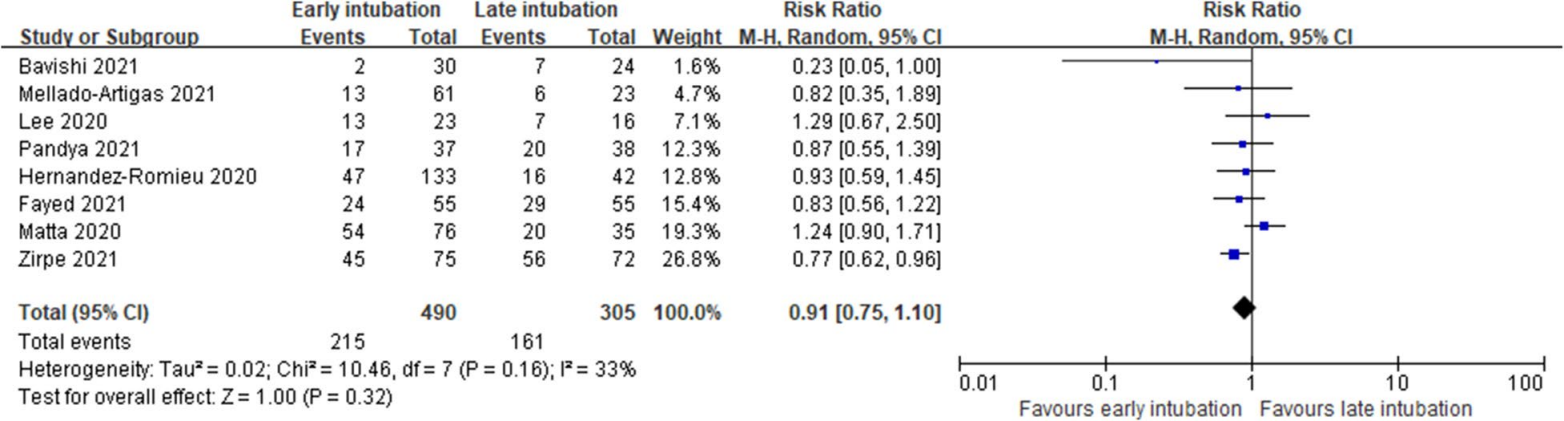
In-hospital mortality

**Fig. 4:**
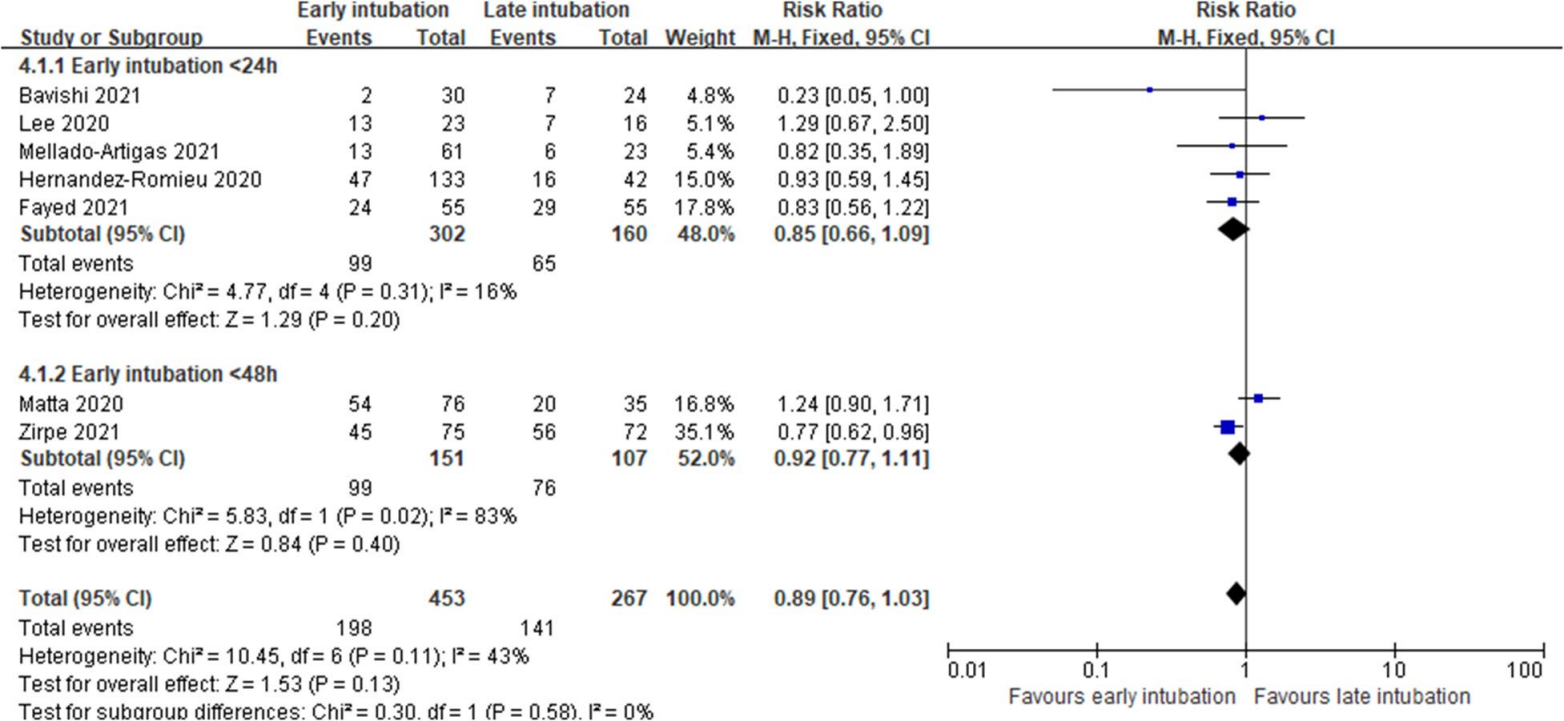
Subgroup analysis of in-hospital mortality by definition of early intubation as < 24 h or < 48 h from index time

**Fig. 5:**
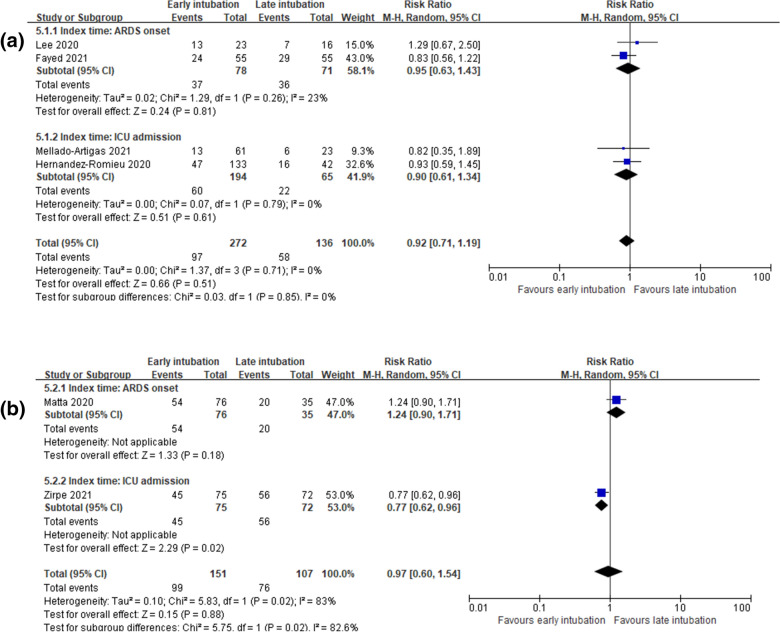
Subgroup analysis of in-hospital mortality by index time in studies defining early intubation as < 24 h (**a**) or < 48 h (**b**). **a** Subgroup analysis of in-hospital mortality by index time in studies defining early intubation as < 24 h. **b** Subgroup analysis of in-hospital mortality by index time in studies defining early intubation as < 48 h

Nine studies reported in-hospital mortality. Of the nine studies, eights studies [14, 15, 17, 26, 28–31] reporting numbers of death were included for quantitative synthesis and one study [32] reporting hazard ratio (HR) was not synthesized. Overall, the incidence of in-hospital mortality was 43.9% (215/490) in the early intubation group and 52.8% (161/305) in the late intubation group. In-hospital mortality was similar between the early and late intubation groups (RR, 0.91; 95% CI 0.75–1.10; *P* = 0.32; *I*^2^ = 33%; very low certainty of evidence; Fig. 3). Consistent results were obtained for subgroup analysis based on the definition of early intubation (< 24 h or < 48 h) (Fig. 4) and the index time (ARDS onset or ICU admission) except for the subgroup intubated before 48 h from the index time of ICU admission (Fig. 5). Parish et al. [32] reported that early intubation was not significantly associated with differences in mortality (HR, 1.09; 95% CI, 0.94 to 1.26; *P* = 0.26).

### Secondary outcomes

#### ICU length of stay

**Fig. 6:**
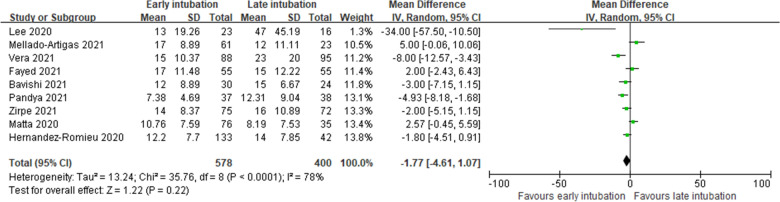
Length of stay in the intensive care unit

Nine studies reported LOS in the ICU [14–17, 26, 28–31]. We found no significant difference according to the timing of intubation (978 patients; MD, −1.77 days; 95% CI −4.61 to 1.07; *P* = 0.22; *I*^2^ = 78%; very low certainty of evidence; Fig. 6).

#### Duration of mechanical ventilation

**Fig. 7:**
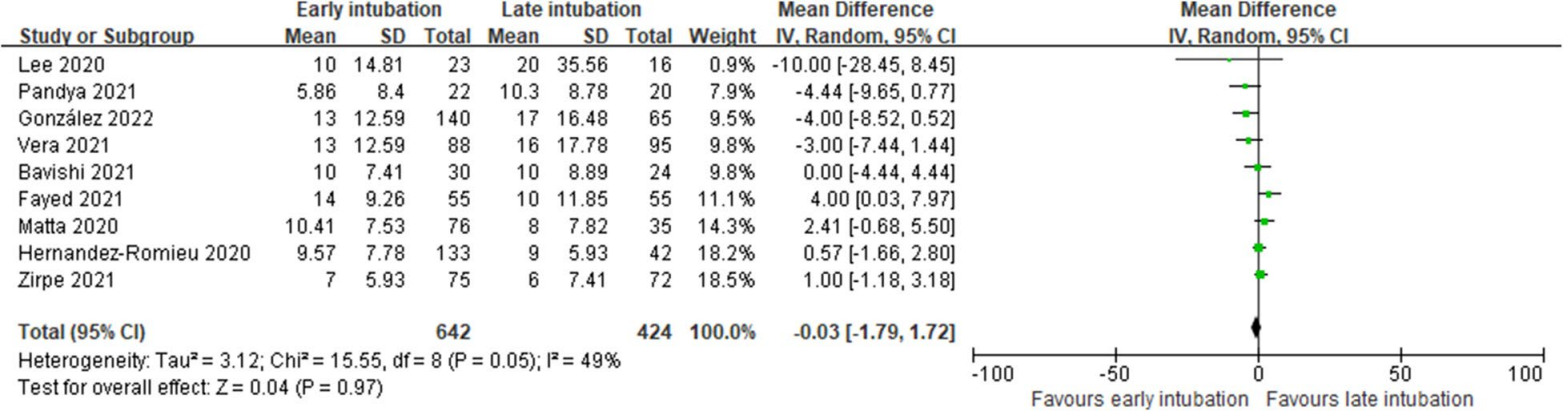
Duration of mechanical ventilation

Nine studies reported the duration of mechanical ventilation [14–17, 26–30]. We found no significant difference according to the timing of intubation (1066 patients; MD, −0.03 days; 95%, CI −1.79 to 1.72; *P* = 0.97; *I*^2^ = 49%; very low certainty of evidence; Fig. 7).

#### Hospital length of stay

Six studies reported hospital LOS [14–16, 26, 27, 30]. Early intubation compared to late intubation reduced the hospital LOS (738 patients; MD, −4.32 days, 95% CI −7.20 to −1.44; *P* = 0.003; *I*^2^ = 45%; very low certainty of evidence; Additional file 4: Fig. S5). However, the subgroup analysis based on the definition of early intubation (< 24 h or < 48 h) showed no difference between early and late intubation (Additional file 4: Fig. S6).

#### ICU-free days and ventilator-free days

One study involving 32 patients reported ICU-free days [33]. Early intubation (median, 0 day; IQR, 0–16 days) was not significantly associated with lesser ICU-free days than that observed for late intubation (median, 0 day; IQR, 0–4 days) (*P* = 0.39). Four studies reported ventilator-free days [16, 29, 31, 33]. We found no evidence of a difference in ventilator-free days according to the timing of intubation (344 patients; MD, 0.94 day; 95% CI −4.56 to 6.43; *P* = 0.74; *I*^2^ = 54%; Additional file 4: Fig. S7).

The GRADE summary of findings table of in-hospital mortality, ICU LOS, duration of mechanical ventilation, and hospital LOS is reported in Table 2.

**Table 2:** GRADE summary of findings table of in-hospital mortality, ICU LOS, duration of mechanical ventilation, and hospital LOS

Outcomes	Anticipated absolute effects (95% CI)		Relative effect (95% CI)	No. of participants (studies)	Certainty of the evidence (GRADE)
	Risk with late intubation	Risk with early intubation			
In-hospital mortality	528 per 1,000	480 per 1,000 (396 to 581)	RR 0.91 (0.75 to 1.10)	795 (8 observational studies)	⨁◯◯◯ Very low^a^
	Parish, et al. [32] reported that early intubation was not significantly associated with differences in in-hospital mortality (hazard ratio 1.09, 95% CI 0.94 to 1.26, P = 0.26)			1,628 (1 observational study)	
ICU length of stay	The mean ICU LOS was 0	MD 1.77 lower (4.61 lower to 1.07 higher)	-	978 (9 observational studies)	⨁◯◯◯ Very low^b,c^
Duration of mechanical ventilation	The mean ventilator duration was 0	MD 0.03 lower (1.79 lower to 1.72 higher)	-	1,066 (9 observational studies)	⨁◯◯◯ Very low^b^
Hospital length of stay	The mean hospital LOS was 0	MD 4.32 lower (7.2 lower to 1.44 lower)	-	738 (6 observational studies)	⨁◯◯◯ Very low^b^

*CI* confidence interval, *MD* mean difference, *RR* relative risk, *ICU* intensive care unit, *LOS* length of stay

*GRADE Working Group grades of evidence*: High certainty: we are very confident that the true effect lies close to that of the estimate of the effect. Moderate certainty: we are moderately confident in the effect estimate: the true effect is likely to be close to the estimate of the effect, but there is a possibility that it is substantially different. Low certainty: our confidence in the effect estimate is limited: the true effect may be substantially different from the estimate of the effect. Very low certainty: we have very little confidence in the effect estimate: the true effect is likely to be substantially different from the estimate of effect

^a^Downgrade for risk of bias concern in the domains of target group selection and confounder

^b^Downgrade for risk of bias concern in the domains of possibility of target group comparisons, target group selection, and confounder

^c^Large I^2^ statistics

## Discussion

In the present study, the key primary outcome, in-hospital mortality, did not differ between the early and late intubation groups. To our knowledge, this study is the most comprehensive meta-analysis of outcomes for the timing of intubation in patients with severe and critical COVID-19 who needed MV. To date, the rationale for early intubation in patients with COVID-19 remains unclear, and individual studies have assessed the appropriate timing of intubation. From the literature reviews, we found that the exact definition of the timing of MV differs across studies. Therefore, we adopted a specific classification in our study for the timing of MV based on the inconsistent results from a previous systematic review performed last year [34]. In addition, more recent studies were included in this review.

The definition of early intubation from the reviewed studies remains unclear. In clinical practice, prediction of clinical deterioration and the time when MV is required for patients is difficult. Moreover, the timing of ICU admission differed according to the nature of the clinical setting in medical facilities. However, the consideration of MV after respiratory failure, including ARDS, is a major predictive factor for ICU admission and potential MV.

According to ICU guidelines, ICU admission is required for patients requiring hourly and/or invasive monitoring or those with respiratory failure considering for MV. Among them, those with ARDS and severe pneumonia are typically admitted to the ICU [35]. As stated in ICU guidelines, despite differences in resources (available clinical expertise, bed availability, etc.), a triage for ICU admission is recommended. As a result, most cases coincide with the time of detection of ARDS and entrance to the ICU.

Management of ARDS is generally supportive, consisting of MV, prevention of stress ulcers and venous thromboembolism, and nutritional support while addressing the underlying etiology. ICU care is usually followed by the detection of ARDS or the potential for ARDS management [36–39]. For instance, a prospective study in Hong Kong investigated the impact on infection control and performance according to intubation time in critically ill patients with COVID-19. Since patients who required 10 L or more of oxygen at baseline were initially eligible and close monitoring was required, early or late intubation was decided after admission to the ICU [40]. As the Spo_2_/Fio_2_ ratio reflects the Pao_2_/Fio_2_ ratio in patients with ARDS, the study population who needed more than 10 L or more oxygen also met the criteria for hypoxemia in ARDS [41]. Moreover, applying NIV or HFNC was proposed in guidelines to be useful in the treatment of ARDS or acute respiratory failure in patients with COVID-19, requiring close monitoring in the ICU and should be progressed to intubation in case of no improvement within 2 h of NIV or HFNC [42, 43]. Therefore, we can assume that the approximating the time between ARDS detection and ICU admission as the primary index for the potential time for MV would be clinically reasonable, considering the COVID-19 pandemic.

However, in our study, we specified the studies that reported the estimation of the timing of intubation from ARDS occurrence [26, 29, 30] or from ICU admission [17, 28, 31]. There was no difference in the results for in-hospital mortality, the primary outcome, even when the index time for ARDS onset or ICU admission was specified in the subgroup analysis (Fig. 5). The strength of this study is that we included studies that reported a clear index time for estimating early or late intubation.

The present study also explored specific clinical outcomes. Heterogeneity among the studies regarding the medical circumstances and pandemic situation was inevitable, resulting in specific consideration of the critical care of patients and utilization of the ICU. In the subgroup analysis of the index time of intubation, there was no difference in in-hospital mortality according to the timing of intubation in critically ill patients with COVID-19. Of these, hospital LOS seemed to be shortened by early intubation, and subgroup analysis based on the definition of early intubation (< 24 h or < 48 h) showed no difference in hospital LOS between early and late intubation.

We also explored studies that reported ICU mortality and 28-day mortality in addition to in-hospital mortality. However, the number of studies that reported ICU [16, 27, 33] and 28-day mortalities [16, 33] was relatively low, and inconsistent indexes were applied in defining early and late intubation: one [33] was from the time of ARDS onset, one [16] was from the time of hospital admission, and another [27] was from the time of first respiratory support. The summarized results for ICU and 28-day mortality rates were difficult to interpret. (Additional file 4: Fig. S8 and Fig. S9).

This study had several limitations. First, relatively few studies were eligible for analysis, and the results showed heterogeneity between the studies. Therefore, further research is required. Second, the results were limited to observational studies because tracheal intubation as an intervention is practically difficult and impossible. Third, the differences in conditions of ICU care according to medical facilities or countries could not be fully considered in the analysis due to the lack of considerable studies in each identified country. Nevertheless, the results of this study will provide important implications for physicians to decide on the timing of intubation for critically ill patients with COVID-19 requiring ICU care.

## Conclusions

This study showed that there was no difference in in-hospital mortality between early and late intubation groups. The secondary outcomes, including ICU LOS, duration of mechanical ventilation, hospital LOS, ICU-free days, and ventilator-free days, did not also differ between the two groups. The decision of physicians who determine the critical care for each critically ill patient with COVID-19 is still important. Further prospective studies would be necessary to support the results with strengthening the certainty of evidence.

## Supplementary Information

**Additional file 1. **PRISMA checklists

**Additional file 2. **Search strategies

**Additional file 3. **List of excluded studies after full-text screening

**Additional file 4. **Forest plots. **Figure S1.** Subgroup analysis of length of stay in ICU by definition of early intubation as < 24 h or < 48 h from index time. **Figure S2.** Subgroup analysis of length of stay in ICU by index time in studies defining early intubation as < 24 h (a) or < 48 h (b). **Figure S3.** Subgroup analysis of duration of mechanical ventilation by definition of early intubation as < 24 h or < 48 h from index time. **Figure S4.** Subgroup analysis of duration of mechanical ventilation by index time in studies defining early intubation as < 24 h (a) or < 48 h (b). **Figure S5.** Hospital length of stay. **Figure S6.** Subgroup analysis of hospital length of stay by definition of early intubation as < 24 h or < 48 h from index time. **Figure S7.** Ventilator-free days. **Figure S8.** ICU mortality. **Figure S9.** 28-day mortality

## Data Availability

The datasets used and/or analyzed during the current study are available from the corresponding author upon reasonable request.

## Abbreviations

ARDS: Acute respiratory distress syndrome
COVID-19: Coronavirus disease 2019
SILI: Prevent self-induced lung injury
CPAP: Continuous positive airway pressure
NIV: Non-invasive ventilation
HFNC: High-flow nasal cannula
MV: Mechanical ventilation
LOS: Length of stay
ICU: Intensive care unit
RoBANS: Risk of Bias Assessment tool for Non-randomized studies
RR: Relative risks
CI: Confidence interval
HR: Hazard ratio
MD: Mean differences
SD: Standard deviation
GRADE: Grading of Recommendations, Assessment, Development and Evaluation
