# Supplementary material for: Early intubation and clinical outcomes in patients with severe COVID-19: a systematic review and meta-analysis
Source: Eur J Med Res. 2022 Nov 3;27:226. doi: 10.1186/s40001-022-00841-6 (PMC9631590; doi:10.1186/s40001-022-00841-6)
Supplement: Supplementary file 2 — Additional file 2. Search strategies [file 40001_2022_841_MOESM2_ESM.docx]

**Additional file 2. Search strategies**

**PubMed**

| No. | Search strategy |
| --- | --- |
| #1 | "COVID-19"[Mesh] |
| #2 | "COVID-19"[TW] OR "COVID 19"[TW] OR "COVID-19 Virus Disease"[TW] OR "COVID 19 Virus Disease"[TW] OR "COVID-19 Virus Diseases"[TW] OR "Disease, COVID-19 Virus"[TW] OR "Virus Disease, COVID-19"[TW] OR "COVID-19 Virus Infection"[TW] OR "COVID 19 Virus Infection"[TW] OR "COVID-19 Virus Infections"[TW] OR "Infection, COVID-19 Virus"[TW] OR "Virus Infection, COVID-19"[TW] OR "2019-nCoV Infection"[TW] OR "2019 nCoV Infection"[TW] OR "2019-nCoV Infections"[TW] OR "Infection, 2019-nCoV"[TW] OR "Coronavirus Disease-19"[TW] OR "Coronavirus Disease 19"[TW] OR "2019-nCoV Disease"[TW] OR "2019 nCoV Disease"[TW] OR "2019-nCoV Diseases"[TW] OR "Disease, 2019-nCoV"[TW] OR "COVID19"[TW] OR "Coronavirus Disease 2019"[TW] OR "Disease 2019, Coronavirus"[TW] OR "SARS Coronavirus 2 Infection"[TW] OR "SARS-CoV-2 Infection"[TW] OR "Infection, SARS-CoV-2"[TW] OR "SARS CoV 2 Infection"[TW] OR "SARS-CoV-2 Infections"[TW] OR "COVID-19 Pandemic"[TW] OR "COVID 19 Pandemic"[TW] OR "COVID-19 Pandemics"[TW] OR "Pandemic, COVID-19"[TW] OR "2019 Novel Coronavirus Disease"[TW] OR "2019 Novel Coronavirus Infection"[TW] OR "coronavirus disease 2"[TW] OR "coronavirus disease 2019 pneumonia"[TW] OR "coronavirus infection 2019"[TW] OR "COVID"[TW] OR "COVID 19 induced pneumonia"[TW] OR "COVID 2019"[TW] OR "COVID-19 induced pneumonia"[TW] OR "COVID-19 pneumonia"[TW] OR "nCoV 2019 disease"[TW] OR "nCoV 2019 infection"[TW] OR "paucisymptomatic coronavirus disease 2019"[TW] OR "SARS coronavirus 2 pneumonia"[TW] OR "SARSCoV2 disease"[TW] OR "SARS-CoV2 disease"[TW] OR "SARS-CoV-2 disease"[TW] OR "SARSCoV2 infection"[TW] OR "SARS-CoV2 infection"[TW] OR "SARS-CoV-2 pneumonia"[TW] OR "severe acute respiratory syndrome 2"[TW] OR "severe acute respiratory syndrome 2 pneumonia"[TW] OR "severe acute respiratory syndrome coronavirus 2 infection"[TW] OR "severe acute respiratory syndrome coronavirus 2019 infection"[TW] OR "severe acute respiratory syndrome CoV-2 infection"[TW] OR "Wuhan coronavirus disease"[TW] OR "Wuhan coronavirus infection"[TW] OR "2019 novel coronavirus epidemic"[TW] OR "new coronavirus pneumonia"[TW] OR "novel coronavirus 2019 disease"[TW] OR "novel coronavirus 2019 infection"[TW] OR "novel coronavirus disease 2019"[TW] OR "novel coronavirus infected pneumonia"[TW] OR "novel coronavirus infection 2019"[TW] OR "novel coronavirus pneumonia"[TW] OR "2019nCoV"[TW] OR "19nCoV"[TW] OR "COVID19*"[TW] OR "SARSCOV-2"[TW] OR "SARSCOV2"[TW] OR "corona virus 2"[TW] OR "Wuhan"[TW] OR "Hubei"[TW] OR "new coronavirus"[TW] OR "novel coronavirus"[TW] OR "novel corona virus"[TW] OR "novel CoV"[TW] |
| #3 | "SARS-CoV-2"[Mesh] |
| #4 | "SARS-CoV-2"[TW] OR "Coronavirus Disease 2019 Virus"[TW] OR "Wuhan Seafood Market Pneumonia Virus"[TW] OR "SARS-CoV-2 Virus"[TW] OR "SARS CoV 2 Virus"[TW] OR "SARS-CoV-2 Viruses"[TW] OR "Virus, SARS-CoV-2"[TW] OR "2019-nCoV"[TW] OR "COVID-19 Virus"[TW] OR "COVID 19 Virus"[TW] OR "COVID-19 Viruses"[TW] OR "Virus, COVID-19"[TW] OR "Wuhan Coronavirus"[TW] OR "Coronavirus, Wuhan"[TW] OR "SARS Coronavirus 2"[TW] OR "Coronavirus 2, SARS"[TW] OR "Severe Acute Respiratory Syndrome Coronavirus 2"[TW] OR "2019 Novel Coronavirus"[TW] OR "2019 Novel Coronaviruses"[TW] OR "Coronavirus, 2019 Novel"[TW] OR "Novel Coronavirus, 2019"[TW] OR "2019 nCOV"[TW] OR "2019 severe acute respiratory syndrome coronavirus 2"[TW] OR "HCoV-19"[TW] OR "Human coronavirus 2019"[TW] OR "nCoV-2019"[TW] OR "SARS2 (virus)"[TW] OR "SARS-related coronavirus 2"[TW] OR "Sever acute respiratory syndrome coronavirus 2"[TW] OR "Severe acute respiratory coronavirus 2"[TW] OR "Severe acute respiratory syndorme coronavirus 2"[TW] OR "severe acute respiratory syndrome 2 virus"[TW] OR "severe acute respiratory syndrome corona virus 2"[TW] OR "severe acute respiratory syndrome coronavirus 2019"[TW] OR "Severe acute respiratory syndrome coronoavirus 2"[TW] OR "Severe acute respiratory syndrome coronvirus 2"[TW] OR "severe acute respiratory syndrome CoV-2 virus"[TW] OR "Severe acute respiratory syndrome related coronavirus 2"[TW] OR "Severe acute respiratory syndrome virus 2"[TW] OR "Severe acute respiratoy syndrome coronavirus 2"[TW] OR "2019 new coronavirus"[TW] OR "novel 2019 coronavirus"[TW] OR "novel coronavirus-19"[TW] |
| #5 | #1 OR #2 OR #3 OR #4 |
| #6 | "Critical Care"[Mesh] OR "Critical Care"[TW] |
| #7 | "Intensive Care units"[Mesh] OR "Intensive Care units"[TW] |
| #8 | "Trauma Centers"[Mesh] OR "Trauma Centers"[TW] |
| #9 | "critical care unit"[TW] OR "intensive care unit"[TW] OR "cardica care unit"[TW] OR "intensive therapy unit"[TW] OR "high-dependency unit"[TW] OR "ICU"[TW] OR "PICU"[TW] OR "MICU"[TW] OR "CICU"[TW] OR "CVICU"[TW] OR "CCU"[TW] OR "NICU"[TW] OR "SICU"[TW] OR "POCCU"[TW] OR "ITU"[TW] OR "HDU"[TW] OR "severe"[TW] OR "critical"[TW] OR "critically ill"[TW] |
| #10 | "high-flow nasal cannula"[TW] OR "high-flow oxygen therapy"[TW] OR "nasal high-flow oxygen therapy"[TW] OR "high flow nasal cannula"[TW] OR "high flow nasal therapy"[TW] OR "high flow nasal oxygen"[TW] OR "high flow oxygen therapy"[TW] OR "high flow therapy"[TW] OR "nasal highflow"[TW] OR "optiflow"[TW] |
| #11 | "Noninvasive Ventilation"[Mesh] OR "Noninvasive Ventilation"[TW] OR "non-invasive positive pressure ventilation"[TW] OR "noninvasive positive pressure ventilation"[TW] OR "non-invasive ventilation"[TW] |
| #12 | "Respiratory Distress Syndrome"[Mesh] |
| #13 | "Respiratory Distress Syndrome"[TW] OR "Acute Respiratory Distress Syndrome"[TW] OR "Severe Respiratory Distress Syndrome"[TW] OR "ARDS"[TW] |
| #14 | "Respiratory Insufficiency"[Mesh] Or "Respiratory Insufficiency"[TW] |
| #15 | ("hypoxia"[TW] OR "hypoxemia"[TW] OR "hypoximic"[TW] OR "hypercapnic"[TW] OR Oxygen Deficienc*[TW]) AND ("lung"[Mesh] OR "lung"[TW] OR "pulmonary"[TW] OR "repiratory"[TW]) |
| #16 | "Acute Lung Injury"[Mesh] |
| #17 | "Acute Lung Injury"[TW] OR "acute lung injury"[TW] OR "respiratory failure"[TW] OR "respiratory insufficiency"[TW] |
| #18 | "Pulmonary Edema"[Mesh] |
| #19 | "Pulmonary Edema"[TW] OR "pulmonary edema"[TW] OR "pulmonary edemas"[TW] |
| #20 | "Pulmonary Fibrosis"[Mesh] |
| #21 | "Pulmonary Fibrosis"[TW] OR "lung fibrosis"[TW] OR "pulmonary fibrosis"[TW] OR "fibrosing alveolitis"[TW] |
| #22 | #6 OR #7 OR #8 OR #9 OR #10 OR #11 OR #12 OR #13 OR #14 OR #15 OR #16 OR #17 OR #18 OR #19 OR #20 OR #21 |
| #23 | "Intubation, Intratracheal"[Mesh] |
| #24 | "Intubation, Intratracheal"[TW] OR "Intubation, Endotracheal"[TW] OR "early intubation"[TW] OR "early endotracheal intubation"[TW] |
| #25 | #23 OR #24 |
| #26 | #5 AND #22 AND #25 |

**Ovid Embase**

| No. | Search strategy |
| --- | --- |
| #1 | exp coronavirus disease 2019/ |
| #2 | ("COVID-19" OR "COVID 19" OR "COVID-19 Virus Disease" OR "COVID 19 Virus Disease" OR "COVID-19 Virus Diseases" OR "Disease, COVID-19 Virus" OR "Virus Disease, COVID-19" OR "COVID-19 Virus Infection" OR "COVID 19 Virus Infection" OR "COVID-19 Virus Infections" OR "Infection, COVID-19 Virus" OR "Virus Infection, COVID-19" OR "2019-nCoV Infection" OR "2019 nCoV Infection" OR "2019-nCoV Infections" OR "Infection, 2019-nCoV" OR "Coronavirus Disease-19" OR "Coronavirus Disease 19" OR "2019-nCoV Disease" OR "2019 nCoV Disease" OR "2019-nCoV Diseases" OR "Disease, 2019-nCoV" OR "COVID19" OR "Coronavirus Disease 2019" OR "Disease 2019, Coronavirus" OR "SARS Coronavirus 2 Infection" OR "SARS-CoV-2 Infection" OR "Infection, SARS-CoV-2" OR "SARS CoV 2 Infection" OR "SARS-CoV-2 Infections" OR "COVID-19 Pandemic" OR "COVID 19 Pandemic" OR "COVID-19 Pandemics" OR "Pandemic, COVID-19" OR "2019 Novel Coronavirus Disease" OR "2019 Novel Coronavirus Infection" OR "coronavirus disease 2" OR "coronavirus disease 2019 pneumonia" OR "coronavirus infection 2019" OR "COVID" OR "COVID 19 induced pneumonia" OR "COVID 2019" OR "COVID-19 induced pneumonia" OR "COVID-19 pneumonia" OR "nCoV 2019 disease" OR "nCoV 2019 infection" OR "paucisymptomatic coronavirus disease 2019" OR "SARS coronavirus 2 pneumonia" OR "SARSCoV2 disease" OR "SARS-CoV2 disease" OR "SARS-CoV-2 disease" OR "SARSCoV2 infection" OR "SARS-CoV2 infection" OR "SARS-CoV-2 pneumonia" OR "severe acute respiratory syndrome 2" OR "severe acute respiratory syndrome 2 pneumonia" OR "severe acute respiratory syndrome coronavirus 2 infection" OR "severe acute respiratory syndrome coronavirus 2019 infection" OR "severe acute respiratory syndrome CoV-2 infection" OR "Wuhan coronavirus disease" OR "Wuhan coronavirus infection" OR "2019 novel coronavirus epidemic" OR "new coronavirus pneumonia" OR "novel coronavirus 2019 disease" OR "novel coronavirus 2019 infection" OR "novel coronavirus disease 2019" OR "novel coronavirus infected pneumonia" OR "novel coronavirus infection 2019" OR "novel coronavirus pneumonia" OR "2019nCoV" OR "19nCoV" OR "COVID19$" OR "SARSCOV-2" OR "SARSCOV2" OR "corona virus 2" OR "Wuhan" OR "Hubei" OR "new coronavirus" OR "novel coronavirus" OR "novel corona virus" OR "novel CoV").ti,ab,kw. |
| #3 | exp Severe acute respiratory syndrome coronavirus 2/ |
| #4 | ("SARS-CoV-2" OR "Coronavirus Disease 2019 Virus" OR "Wuhan Seafood Market Pneumonia Virus" OR "SARS-CoV-2 Virus" OR "SARS CoV 2 Virus" OR "SARS-CoV-2 Viruses" OR "Virus, SARS-CoV-2" OR "2019-nCoV" OR "COVID-19 Virus" OR "COVID 19 Virus" OR "COVID-19 Viruses" OR "Virus, COVID-19" OR "Wuhan Coronavirus" OR "Coronavirus, Wuhan" OR "SARS Coronavirus 2" OR "Coronavirus 2, SARS" OR "Severe Acute Respiratory Syndrome Coronavirus 2" OR "2019 Novel Coronavirus" OR "2019 Novel Coronaviruses" OR "Coronavirus, 2019 Novel" OR "Novel Coronavirus, 2019" OR "2019 nCOV" OR "2019 severe acute respiratory syndrome coronavirus 2" OR "HCoV-19" OR "Human coronavirus 2019" OR "nCoV-2019" OR "SARS2 (virus)" OR "SARS-related coronavirus 2" OR "Sever acute respiratory syndrome coronavirus 2" OR "Severe acute respiratory coronavirus 2" OR "Severe acute respiratory syndorme coronavirus 2" OR "severe acute respiratory syndrome 2 virus" OR "severe acute respiratory syndrome corona virus 2" OR "severe acute respiratory syndrome coronavirus 2019" OR "Severe acute respiratory syndrome coronoavirus 2" OR "Severe acute respiratory syndrome coronvirus 2" OR "severe acute respiratory syndrome CoV-2 virus" OR "Severe acute respiratory syndrome related coronavirus 2" OR "Severe acute respiratory syndrome virus 2" OR "Severe acute respiratoy syndrome coronavirus 2" OR "2019 new coronavirus" OR "novel 2019 coronavirus" OR "novel coronavirus-19").ti,ab,kw. |
| #5 | 1 or 2 or 3 or 4 |
| #6 | exp intensive care/ OR "Critical Care".ti,ab,kw. |
| #7 | exp intensive care unit/ OR "Intensive Care units".ti,ab,kw. |
| #8 | exp emergency health service/ OR "Trauma Centers".ti,ab,kw. |
| #9 | ("critical care unit" OR "intensive care unit" OR "cardica care unit" OR "intensive therapy unit" OR "high-dependency unit" OR "ICU" OR "PICU" OR "MICU" OR "CICU" OR "CVICU" OR "CCU" OR "NICU" OR "SICU" OR "POCCU" OR "ITU" OR "HDU" OR "severe" OR "critical" OR "critically ill").ti,ab,kw. |
| #10 | ("high-flow nasal cannula" OR "high-flow oxygen therapy" OR "nasal high-flow oxygen therapy" OR "high flow nasal cannula" OR "high flow nasal therapy" OR "high flow nasal oxygen" OR "high flow oxygen therapy" OR "high flow therapy" OR "nasal highflow" OR "optiflow").ti,ab,kw. |
| #11 | exp noninvasive ventilation/ OR ("Noninvasive Ventilation" OR "non-invasive positive pressure ventilation" OR "noninvasive positive pressure ventilation" OR "non-invasive ventilation").ti,ab,kw. |
| #12 | exp respiratory distress syndrome/ |
| #13 | ("Respiratory Distress Syndrome" OR "Acute Respiratory Distress Syndrome" OR "Severe Respiratory Distress Syndrome" OR "ARDS").ti,ab,kw. |
| #14 | exp respiratory failure/ OR "Respiratory Insufficiency".ti,ab,kw. |
| #15 | (("hypoxia" OR "hypoxemia" OR "hypoximic" OR "hypercapnic" OR Oxygen Deficienc*).ti,ab,kw.) AND (exp lung/ OR ("lung" OR "pulmonary" OR "repiratory").ti,ab,kw.) |
| #16 | exp acute lung injury/ |
| #17 | ("Acute Lung Injury" OR "acute lung injury" OR "respiratory failure" OR "respiratory insufficiency").ti,ab,kw. |
| #18 | exp lung edema/ |
| #19 | ("Pulmonary Edema" OR "pulmonary edema" OR "pulmonary edemas").ti,ab,kw. |
| #20 | exp lung fibrosis/ |
| #21 | ("Pulmonary Fibrosis" OR "lung fibrosis" OR "pulmonary fibrosis" OR "fibrosing alveolitis").ti,ab,kw. |
| #22 | 6 or 7 or 8 or 9 or 10 or 11 or 12 or 13 or 14 or 15 or 16 or 17 or 18 or 19 or 20 or 21 |
| #23 | exp endotracheal intubation/ |
| #24 | ("Intubation, Intratracheal" OR "Intubation, Endotracheal" OR "early intubation" OR "ealy endotracheal intubation").ti,ab,kw. |
| #25 | 23 or 24 |
| #26 | 5 and 22 and 25 |

**Cochrane Library**

| No. | Search strategy |
| --- | --- |
| #1 | [mh "COVID-19"] |
| #2 | "COVID-19":ti,ab,kw OR "COVID 19":ti,ab,kw OR "COVID-19 Virus Disease":ti,ab,kw OR "COVID 19 Virus Disease":ti,ab,kw OR "COVID-19 Virus Diseases":ti,ab,kw OR "Disease, COVID-19 Virus":ti,ab,kw OR "Virus Disease, COVID-19":ti,ab,kw OR "COVID-19 Virus Infection":ti,ab,kw OR "COVID 19 Virus Infection":ti,ab,kw OR "COVID-19 Virus Infections":ti,ab,kw OR "Infection, COVID-19 Virus":ti,ab,kw OR "Virus Infection, COVID-19":ti,ab,kw OR "2019-nCoV Infection":ti,ab,kw OR "2019 nCoV Infection":ti,ab,kw OR "2019-nCoV Infections":ti,ab,kw OR "Infection, 2019-nCoV":ti,ab,kw OR "Coronavirus Disease-19":ti,ab,kw OR "Coronavirus Disease 19":ti,ab,kw OR "2019-nCoV Disease":ti,ab,kw OR "2019 nCoV Disease":ti,ab,kw OR "2019-nCoV Diseases":ti,ab,kw OR "Disease, 2019-nCoV":ti,ab,kw OR "COVID19":ti,ab,kw OR "Coronavirus Disease 2019":ti,ab,kw OR "Disease 2019, Coronavirus":ti,ab,kw OR "SARS Coronavirus 2 Infection":ti,ab,kw OR "SARS-CoV-2 Infection":ti,ab,kw OR "Infection, SARS-CoV-2":ti,ab,kw OR "SARS CoV 2 Infection":ti,ab,kw OR "SARS-CoV-2 Infections":ti,ab,kw OR "COVID-19 Pandemic":ti,ab,kw OR "COVID 19 Pandemic":ti,ab,kw OR "COVID-19 Pandemics":ti,ab,kw OR "Pandemic, COVID-19":ti,ab,kw OR "2019 Novel Coronavirus Disease":ti,ab,kw OR "2019 Novel Coronavirus Infection":ti,ab,kw OR "coronavirus disease 2":ti,ab,kw OR "coronavirus disease 2019 pneumonia":ti,ab,kw OR "coronavirus infection 2019":ti,ab,kw OR "COVID":ti,ab,kw OR "COVID 19 induced pneumonia":ti,ab,kw OR "COVID 2019":ti,ab,kw OR "COVID-19 induced pneumonia":ti,ab,kw OR "COVID-19 pneumonia":ti,ab,kw OR "nCoV 2019 disease":ti,ab,kw OR "nCoV 2019 infection":ti,ab,kw OR "paucisymptomatic coronavirus disease 2019":ti,ab,kw OR "SARS coronavirus 2 pneumonia":ti,ab,kw OR "SARSCoV2 disease":ti,ab,kw OR "SARS-CoV2 disease":ti,ab,kw OR "SARS-CoV-2 disease":ti,ab,kw OR "SARSCoV2 infection":ti,ab,kw OR "SARS-CoV2 infection":ti,ab,kw OR "SARS-CoV-2 pneumonia":ti,ab,kw OR "severe acute respiratory syndrome 2":ti,ab,kw OR "severe acute respiratory syndrome 2 pneumonia":ti,ab,kw OR "severe acute respiratory syndrome coronavirus 2 infection":ti,ab,kw OR "severe acute respiratory syndrome coronavirus 2019 infection":ti,ab,kw OR "severe acute respiratory syndrome CoV-2 infection":ti,ab,kw OR "Wuhan coronavirus disease":ti,ab,kw OR "Wuhan coronavirus infection":ti,ab,kw OR "2019 novel coronavirus epidemic":ti,ab,kw OR "new coronavirus pneumonia":ti,ab,kw OR "novel coronavirus 2019 disease":ti,ab,kw OR "novel coronavirus 2019 infection":ti,ab,kw OR "novel coronavirus disease 2019":ti,ab,kw OR "novel coronavirus infected pneumonia":ti,ab,kw OR "novel coronavirus infection 2019":ti,ab,kw OR "novel coronavirus pneumonia":ti,ab,kw OR "2019nCoV":ti,ab,kw OR "19nCoV":ti,ab,kw OR "COVID19*":ti,ab,kw OR "SARSCOV-2":ti,ab,kw OR "SARSCOV2":ti,ab,kw OR "corona virus 2":ti,ab,kw OR "Wuhan":ti,ab,kw OR "Hubei":ti,ab,kw OR "new coronavirus":ti,ab,kw OR "novel coronavirus":ti,ab,kw OR "novel corona virus":ti,ab,kw OR "novel CoV":ti,ab,kw |
| #3 | [mh "SARS-CoV-2"] |
| #4 | "SARS-CoV-2":ti,ab,kw OR "Coronavirus Disease 2019 Virus":ti,ab,kw OR "Wuhan Seafood Market Pneumonia Virus":ti,ab,kw OR "SARS-CoV-2 Virus":ti,ab,kw OR "SARS CoV 2 Virus":ti,ab,kw OR "SARS-CoV-2 Viruses":ti,ab,kw OR "Virus, SARS-CoV-2":ti,ab,kw OR "2019-nCoV":ti,ab,kw OR "COVID-19 Virus":ti,ab,kw OR "COVID 19 Virus":ti,ab,kw OR "COVID-19 Viruses":ti,ab,kw OR "Virus, COVID-19":ti,ab,kw OR "Wuhan Coronavirus":ti,ab,kw OR "Coronavirus, Wuhan":ti,ab,kw OR "SARS Coronavirus 2":ti,ab,kw OR "Coronavirus 2, SARS":ti,ab,kw OR "Severe Acute Respiratory Syndrome Coronavirus 2":ti,ab,kw OR "2019 Novel Coronavirus":ti,ab,kw OR "2019 Novel Coronaviruses":ti,ab,kw OR "Coronavirus, 2019 Novel":ti,ab,kw OR "Novel Coronavirus, 2019":ti,ab,kw OR "2019 nCOV":ti,ab,kw OR "2019 severe acute respiratory syndrome coronavirus 2":ti,ab,kw OR "HCoV-19":ti,ab,kw OR "Human coronavirus 2019":ti,ab,kw OR "nCoV-2019":ti,ab,kw OR "SARS2 (virus)":ti,ab,kw OR "SARS-related coronavirus 2":ti,ab,kw OR "Sever acute respiratory syndrome coronavirus 2":ti,ab,kw OR "Severe acute respiratory coronavirus 2":ti,ab,kw OR "Severe acute respiratory syndorme coronavirus 2":ti,ab,kw OR "severe acute respiratory syndrome 2 virus":ti,ab,kw OR "severe acute respiratory syndrome corona virus 2":ti,ab,kw OR "severe acute respiratory syndrome coronavirus 2019":ti,ab,kw OR "Severe acute respiratory syndrome coronoavirus 2":ti,ab,kw OR "Severe acute respiratory syndrome coronvirus 2":ti,ab,kw OR "severe acute respiratory syndrome CoV-2 virus":ti,ab,kw OR "Severe acute respiratory syndrome related coronavirus 2":ti,ab,kw OR "Severe acute respiratory syndrome virus 2":ti,ab,kw OR "Severe acute respiratoy syndrome coronavirus 2":ti,ab,kw OR "2019 new coronavirus":ti,ab,kw OR "novel 2019 coronavirus":ti,ab,kw OR "novel coronavirus-19":ti,ab,kw |
| #5 | #1 OR #2 OR #3 OR #4 |
| #6 | [mh "Critical Care"] OR "Critical Care":ti,ab,kw |
| #7 | [mh "Intensive Care units"] OR "Intensive Care units":ti,ab,kw |
| #8 | [mh "Trauma Centers"] OR "Trauma Centers":ti,ab,kw |
| #9 | "critical care unit":ti,ab,kw OR "intensive care unit":ti,ab,kw OR "cardica care unit":ti,ab,kw OR "intensive therapy unit":ti,ab,kw OR "high-dependency unit":ti,ab,kw OR "ICU":ti,ab,kw OR "PICU":ti,ab,kw OR "MICU":ti,ab,kw OR "CICU":ti,ab,kw OR "CVICU":ti,ab,kw OR "CCU":ti,ab,kw OR "NICU":ti,ab,kw OR "SICU":ti,ab,kw OR "POCCU":ti,ab,kw OR "ITU":ti,ab,kw OR "HDU":ti,ab,kw OR "severe":ti,ab,kw OR "critical":ti,ab,kw OR "critically ill":ti,ab,kw |
| #10 | "high-flow nasal cannula":ti,ab,kw OR "high-flow oxygen therapy":ti,ab,kw OR "nasal high-flow oxygen therapy":ti,ab,kw OR "high flow nasal cannula":ti,ab,kw OR "high flow nasal therapy":ti,ab,kw OR "high flow nasal oxygen":ti,ab,kw OR "high flow oxygen therapy":ti,ab,kw OR "high flow therapy":ti,ab,kw OR "nasal highflow":ti,ab,kw OR "optiflow":ti,ab,kw |
| #11 | [mh "Noninvasive Ventilation"] OR "Noninvasive Ventilation":ti,ab,kw OR "non-invasive positive pressure ventilation":ti,ab,kw OR "noninvasive positive pressure ventilation":ti,ab,kw OR "non-invasive ventilation":ti,ab,kw |
| #12 | [mh "Respiratory Distress Syndrome"] |
| #13 | "Respiratory Distress Syndrome":ti,ab,kw OR "Acute Respiratory Distress Syndrome":ti,ab,kw OR "Severe Respiratory Distress Syndrome":ti,ab,kw OR "ARDS":ti,ab,kw |
| #14 | [mh "Respiratory Insufficiency"] OR "Respiratory Insufficiency":ti,ab,kw |
| #15 | ("hypoxia":ti,ab,kw OR "hypoxemia":ti,ab,kw OR "hypoximic":ti,ab,kw OR "hypercapnic":ti,ab,kw OR Oxygen Deficienc*:ti,ab,kw) AND ([mh "lung"] OR "lung":ti,ab,kw OR "pulmonary":ti,ab,kw OR "repiratory":ti,ab,kw) |
| #16 | [mh "Acute Lung Injury"] |
| #17 | "Acute Lung Injury":ti,ab,kw OR "acute lung injury":ti,ab,kw OR "respiratory failure":ti,ab,kw OR "respiratory insufficiency":ti,ab,kw |
| #18 | [mh "Pulmonary Edema"] |
| #19 | "Pulmonary Edema":ti,ab,kw OR "pulmonary edema":ti,ab,kw OR "pulmonary edemas":ti,ab,kw |
| #20 | [mh "Pulmonary Fibrosis"] |
| #21 | "Pulmonary Fibrosis":ti,ab,kw OR "lung fibrosis":ti,ab,kw OR "pulmonary fibrosis":ti,ab,kw OR "fibrosing alveolitis":ti,ab,kw |
| #22 | #6 OR #7 OR #8 OR #9 OR #10 OR #11 OR #12 OR #13 OR #14 OR #15 OR #16 OR #17 OR #18 OR #19 OR #20 OR #21 |
| #23 | [mh "Intubation, Intratracheal"] |
| #24 | "Intubation, Intratracheal":ti,ab,kw OR "Intubation, Endotracheal":ti,ab,kw OR "early intubation":ti,ab,kw OR "ealy endotracheal intubation":ti,ab,kw |
| #25 | #23 OR #24 |
| #26 | #5 AND #22 AND #25 |

**KMBASE**

| No. | Search strategy |
| --- | --- |
| #1 | (((([ALL=COVID-19] OR [ALL=COVID19]) OR [ALL=coronavirus]) AND (((([ALL=Critical] OR [ALL=Intensive]) OR [ALL=Severe]) OR [ALL=Trauma Centers]) OR [ALL=Critically ill])) AND (([ALL=Intratracheal Intubation] OR [ALL=Endotracheal Intubation]) OR [ALL=early intubation])) |
| #2 | (((([ALL=COVID-19] OR [ALL=COVID19]) OR [ALL=coronavirus]) AND (((([ALL=Respiratory] OR [ALL=ARDS]) OR [ALL=lung]) OR [ALL=pulmonary]) OR [ALL=fibrosing alveolitis])) AND (([ALL=Intratracheal Intubation] OR [ALL=Endotracheal Intubation]) OR [ALL=early intubation])) |
| #3 | (((([ALL=COVID-19] OR [ALL=COVID19]) OR [ALL=coronavirus]) AND (((([ALL=pneumothorax] OR [ALL=Mediastinal Emphysema]) OR [ALL=Pneumomediastinum]) OR [ALL=hypoxia]) OR [ALL=hypoxemia])) AND (([ALL=Intratracheal Intubation] OR [ALL=Endotracheal Intubation]) OR [ALL=early intubation])) |
| #4 | (((([ALL=COVID-19] OR [ALL=COVID19]) OR [ALL=coronavirus]) AND (([ALL=HFNC] OR [ALL=high flow]) OR [ALL=optiflow])) AND (([ALL=Intratracheal Intubation] OR [ALL=Endotracheal Intubation]) OR [ALL=early intubation])) |
| #5 | (((([ALL=COVID-19] OR [ALL=COVID19]) OR [ALL=coronavirus]) AND ([ALL=NIPPV] OR [ALL=non-invasive positive pressure])) AND (([ALL=Intratracheal Intubation] OR [ALL=Endotracheal Intubation]) OR [ALL=early intubation])) |
| #6 | (((([ALL=COVID-19] OR [ALL=COVID19]) OR [ALL=coronavirus]) AND ([ALL=BIPAP] OR [ALL=noninvasive positive pressure])) AND (([ALL=Intratracheal Intubation] OR [ALL=Endotracheal Intubation]) OR [ALL=early intubation])) |
| #7 | (((([ALL=COVID-19] OR [ALL=COVID19]) OR [ALL=coronavirus]) AND ([ALL=non-invasive ventilation] OR [ALL=noninvasive ventilation])) AND (([ALL=Intratracheal Intubation] OR [ALL=Endotracheal Intubation]) OR [ALL=early intubation])) |
| #8 | (((([ALL=COVID-19] OR [ALL=COVID19]) OR [ALL=coronavirus]) AND [ALL=Intermittent Positive-Pressure Ventilation]) AND (([ALL=Intratracheal Intubation] OR [ALL=Endotracheal Intubation]) OR [ALL=early intubation])) |
| #9 | (([ALL=SARS-CoV-2] AND (((([ALL=Critical] OR [ALL=Intensive]) OR [ALL=Severe]) OR [ALL=Trauma Centers]) OR [ALL=Critically ill])) AND (([ALL=Intratracheal Intubation] OR [ALL=Endotracheal Intubation]) OR [ALL=early intubation])) |
| #10 | (([ALL=Severe acute respiratory syndrome coronavirus 2] AND (((([ALL=Critical] OR [ALL=Intensive]) OR [ALL=Severe]) OR [ALL=Trauma Centers]) OR [ALL=Critically ill])) AND (([ALL=Intratracheal Intubation] OR [ALL=Endotracheal Intubation]) OR [ALL=early intubation])) |
| #11 | (([ALL=SARS-CoV-2] AND (((([ALL=Respiratory] OR [ALL=ARDS]) OR [ALL=lung]) OR [ALL=pulmonary]) OR [ALL=fibrosing alveolitis])) AND (([ALL=Intratracheal Intubation] OR [ALL=Endotracheal Intubation]) OR [ALL=early intubation])) |
| #12 | (([ALL=Severe acute respiratory syndrome coronavirus 2] AND (((([ALL=Respiratory] OR [ALL=ARDS]) OR [ALL=lung]) OR [ALL=pulmonary]) OR [ALL=fibrosing alveolitis])) AND (([ALL=Intratracheal Intubation] OR [ALL=Endotracheal Intubation]) OR [ALL=early intubation])) |
| #13 | (([ALL=SARS-CoV-2] AND (((([ALL=pneumothorax] OR [ALL=Mediastinal Emphysema]) OR [ALL=Pneumomediastinum]) OR [ALL=hypoxia]) OR [ALL=hypoxemia])) AND (([ALL=Intratracheal Intubation] OR [ALL=Endotracheal Intubation]) OR [ALL=early intubation])) |
| #14 | (([ALL=Severe acute respiratory syndrome coronavirus 2] AND (((([ALL=pneumothorax] OR [ALL=Mediastinal Emphysema]) OR [ALL=Pneumomediastinum]) OR [ALL=hypoxia]) OR [ALL=hypoxemia])) AND (([ALL=Intratracheal Intubation] OR [ALL=Endotracheal Intubation]) OR [ALL=early intubation])) |
| #15 | (([ALL=SARS-CoV-2] AND (([ALL=HFNC] OR [ALL=high flow]) OR [ALL=optiflow])) AND (([ALL=Intratracheal Intubation] OR [ALL=Endotracheal Intubation]) OR [ALL=early intubation])) |
| #16 | (([ALL=Severe acute respiratory syndrome coronavirus 2] AND (([ALL=HFNC] OR [ALL=high flow]) OR [ALL=optiflow])) AND (([ALL=Intratracheal Intubation] OR [ALL=Endotracheal Intubation]) OR [ALL=early intubation])) |
| #17 | (([ALL=SARS-CoV-2] AND ([ALL=NIPPV] OR [ALL=non-invasive positive pressure])) AND (([ALL=Intratracheal Intubation] OR [ALL=Endotracheal Intubation]) OR [ALL=early intubation])) |
| #18 | (([ALL=Severe acute respiratory syndrome coronavirus 2] AND ([ALL=NIPPV] OR [ALL=non-invasive positive pressure])) AND (([ALL=Intratracheal Intubation] OR [ALL=Endotracheal Intubation]) OR [ALL=early intubation])) |
| #19 | (([ALL=SARS-CoV-2] AND ([ALL=BIPAP] OR [ALL=noninvasive positive pressure])) AND (([ALL=Intratracheal Intubation] OR [ALL=Endotracheal Intubation]) OR [ALL=early intubation])) |
| #20 | (([ALL=Severe acute respiratory syndrome coronavirus 2] AND ([ALL=BIPAP] OR [ALL=noninvasive positive pressure])) AND (([ALL=Intratracheal Intubation] OR [ALL=Endotracheal Intubation]) OR [ALL=early intubation])) |
| #21 | (([ALL=SARS-CoV-2] AND ([ALL=non-invasive ventilation] OR [ALL=noninvasive ventilation])) AND (([ALL=Intratracheal Intubation] OR [ALL=Endotracheal Intubation]) OR [ALL=early intubation])) |
| #22 | (([ALL=Severe acute respiratory syndrome coronavirus 2] AND ([ALL=non-invasive ventilation] OR [ALL=noninvasive ventilation])) AND (([ALL=Intratracheal Intubation] OR [ALL=Endotracheal Intubation]) OR [ALL=early intubation])) |
| #23 | (([ALL=SARS-CoV-2] AND [ALL=Intermittent Positive-Pressure Ventilation]) AND (([ALL=Intratracheal Intubation] OR [ALL=Endotracheal Intubation]) OR [ALL=early intubation])) |
| #24 | (([ALL=Severe acute respiratory syndrome coronavirus 2] AND [ALL=Intermittent Positive-Pressure Ventilation]) AND (([ALL=Intratracheal Intubation] OR [ALL=Endotracheal Intubation]) OR [ALL=early intubation])) |
| #25 | (((([ALL=COVID-19] OR [ALL=COVID19]) OR [ALL=coronavirus]) AND (((([ALL=Critical] OR [ALL=Intensive]) OR [ALL=Severe]) OR [ALL=Trauma Centers]) OR [ALL=Critically ill])) AND (([ALL=기관 내 삽관] OR [ALL=기관내삽관]) OR [ALL=조기 삽관])) |
| #26 | (((([ALL=COVID-19] OR [ALL=COVID19]) OR [ALL=coronavirus]) AND (((([ALL=Respiratory] OR [ALL=ARDS]) OR [ALL=lung]) OR [ALL=pulmonary]) OR [ALL=fibrosing alveolitis])) AND (([ALL=기관 내 삽관] OR [ALL=기관내삽관]) OR [ALL=조기 삽관])) |
| #27 | (((([ALL=COVID-19] OR [ALL=COVID19]) OR [ALL=coronavirus]) AND (((([ALL=pneumothorax] OR [ALL=Mediastinal Emphysema]) OR [ALL=Pneumomediastinum]) OR [ALL=hypoxia]) OR [ALL=hypoxemia])) AND (([ALL=기관 내 삽관] OR [ALL=기관내삽관]) OR [ALL=조기 삽관])) |
| #28 | (((([ALL=COVID-19] OR [ALL=COVID19]) OR [ALL=coronavirus]) AND (([ALL=HFNC] OR [ALL=high flow]) OR [ALL=optiflow])) AND (([ALL=기관 내 삽관] OR [ALL=기관내삽관]) OR [ALL=조기 삽관])) |
| #29 | (((([ALL=COVID-19] OR [ALL=COVID19]) OR [ALL=coronavirus]) AND [ALL=NIPPV]) AND (([ALL=기관 내 삽관] OR [ALL=기관내삽관]) OR [ALL=조기 삽관])) |
| #30 | (((([ALL=COVID-19] OR [ALL=COVID19]) OR [ALL=coronavirus]) AND [ALL=non invasive positive pressure]) AND (([ALL=기관 내 삽관] OR [ALL=기관내삽관]) OR [ALL=조기 삽관])) |
| #31 | (((([ALL=COVID-19] OR [ALL=COVID19]) OR [ALL=coronavirus]) AND ([ALL=BIPAP] OR [ALL=noninvasive positive pressure])) AND (([ALL=기관 내 삽관] OR [ALL=기관내삽관]) OR [ALL=조기 삽관])) |
| #32 | (((([ALL=COVID-19] OR [ALL=COVID19]) OR [ALL=coronavirus]) AND ([ALL=non-invasive ventilation] OR [ALL=noninvasive ventilation])) AND (([ALL=기관 내 삽관] OR [ALL=기관내삽관]) OR [ALL=조기 삽관])) |
| #33 | (((([ALL=COVID-19] OR [ALL=COVID19]) OR [ALL=coronavirus]) AND [ALL=Intermittent Positive-Pressure Ventilation]) AND (([ALL=기관 내 삽관] OR [ALL=기관내삽관]) OR [ALL=조기 삽관])) |
| #34 | (([ALL=SARS-CoV-2] AND (((([ALL=Critical] OR [ALL=Intensive]) OR [ALL=Severe]) OR [ALL=Trauma Centers]) OR [ALL=Critically ill])) AND (([ALL=기관 내 삽관] OR [ALL=기관내삽관]) OR [ALL=조기 삽관])) |
| #35 | (([ALL=Severe acute respiratory syndrome coronavirus 2] AND (((([ALL=Critical] OR [ALL=Intensive]) OR [ALL=Severe]) OR [ALL=Trauma Centers]) OR [ALL=Critically ill])) AND (([ALL=기관 내 삽관] OR [ALL=기관내삽관]) OR [ALL=조기 삽관])) |
| #36 | (([ALL=SARS-CoV-2] AND (((([ALL=Respiratory] OR [ALL=ARDS]) OR [ALL=lung]) OR [ALL=pulmonary]) OR [ALL=fibrosing alveolitis])) AND (([ALL=기관 내 삽관] OR [ALL=기관내삽관]) OR [ALL=조기 삽관])) |
| #37 | (([ALL=Severe acute respiratory syndrome coronavirus 2] AND (((([ALL=Respiratory] OR [ALL=ARDS]) OR [ALL=lung]) OR [ALL=pulmonary]) OR [ALL=fibrosing alveolitis])) AND (([ALL=기관 내 삽관] OR [ALL=기관내삽관]) OR [ALL=조기 삽관])) |
| #38 | (([ALL=SARS-CoV-2] AND (((([ALL=pneumothorax] OR [ALL=Mediastinal Emphysema]) OR [ALL=Pneumomediastinum]) OR [ALL=hypoxia]) OR [ALL=hypoxemia])) AND (([ALL=기관 내 삽관] OR [ALL=기관내삽관]) OR [ALL=조기 삽관])) |
| #39 | (([ALL=Severe acute respiratory syndrome coronavirus 2] AND (((([ALL=pneumothorax] OR [ALL=Mediastinal Emphysema]) OR [ALL=Pneumomediastinum]) OR [ALL=hypoxia]) OR [ALL=hypoxemia])) AND (([ALL=기관 내 삽관] OR [ALL=기관내삽관]) OR [ALL=조기 삽관])) |
| #40 | (([ALL=SARS-CoV-2] AND (([ALL=HFNC] OR [ALL=high flow]) OR [ALL=optiflow])) AND (([ALL=기관 내 삽관] OR [ALL=기관내삽관]) OR [ALL=조기 삽관])) |
| #41 | (([ALL=Severe acute respiratory syndrome coronavirus 2] AND (([ALL=HFNC] OR [ALL=high flow]) OR [ALL=optiflow])) AND (([ALL=기관 내 삽관] OR [ALL=기관내삽관]) OR [ALL=조기 삽관])) |
| #42 | (([ALL=SARS-CoV-2] AND ([ALL=NIPPV] OR [ALL=non-invasive positive pressure])) AND (([ALL=기관 내 삽관] OR [ALL=기관내삽관]) OR [ALL=조기 삽관])) |
| #43 | (([ALL=Severe acute respiratory syndrome coronavirus 2] AND ([ALL=NIPPV] OR [ALL=non-invasive positive pressure])) AND (([ALL=기관 내 삽관] OR [ALL=기관내삽관]) OR [ALL=조기 삽관])) |
| #44 | (([ALL=SARS-CoV-2] AND ([ALL=BIPAP] OR [ALL=noninvasive positive pressure])) AND (([ALL=기관 내 삽관] OR [ALL=기관내삽관]) OR [ALL=조기 삽관])) |
| #45 | (([ALL=Severe acute respiratory syndrome coronavirus 2] AND ([ALL=BIPAP] OR [ALL=noninvasive positive pressure])) AND (([ALL=기관 내 삽관] OR [ALL=기관내삽관]) OR [ALL=조기 삽관])) |
| #46 | (([ALL=SARS-CoV-2] AND ([ALL=non-invasive ventilation] OR [ALL=noninvasive ventilation])) AND (([ALL=기관 내 삽관] OR [ALL=기관내삽관]) OR [ALL=조기 삽관])) |
| #47 | (([ALL=Severe acute respiratory syndrome coronavirus 2] AND ([ALL=non-invasive ventilation] OR [ALL=noninvasive ventilation])) AND (([ALL=기관 내 삽관] OR [ALL=기관내삽관]) OR [ALL=조기 삽관])) |
| #48 | (([ALL=SARS-CoV-2] AND [ALL=Intermittent Positive-Pressure Ventilation]) AND (([ALL=기관 내 삽관] OR [ALL=기관내삽관]) OR [ALL=조기 삽관])) |
| #49 | (([ALL=Severe acute respiratory syndrome coronavirus 2] AND [ALL=Intermittent Positive-Pressure Ventilation]) AND (([ALL=기관 내 삽관] OR [ALL=기관내삽관]) OR [ALL=조기 삽관])) |
| #50 | (((([ALL=COVID-19] OR [ALL=COVID19]) OR [ALL=coronavirus]) AND ((((((((([ALL=중환자] OR [ALL=중증]) OR [ALL=호흡부전]) OR [ALL=호흡곤란]) OR [ALL=폐렴]) OR [ALL=폐부종]) OR [ALL=폐섬유증]) OR [ALL=기흉]) OR [ALL=종격기종]) OR [ALL=기종격])) AND (([ALL=Intratracheal Intubation] OR [ALL=Endotracheal Intubation]) OR [ALL=early intubation])) |
| #51 | (((([ALL=COVID-19] OR [ALL=COVID19]) OR [ALL=coronavirus]) AND (([ALL=저산소증] OR [ALL=산소 부족]) OR [ALL=산소결핍])) AND (([ALL=Intratracheal Intubation] OR [ALL=Endotracheal Intubation]) OR [ALL=early intubation])) |
| #52 | (((([ALL=COVID-19] OR [ALL=COVID19]) OR [ALL=coronavirus]) AND (((([ALL=고유량] OR [ALL=비침습적 환기]) OR [ALL=비침습적 양압환기]) OR [ALL=간헐적 양압환기]) OR [ALL=간헐적 양압호흡])) AND (([ALL=Intratracheal Intubation] OR [ALL=Endotracheal Intubation]) OR [ALL=early intubation])) |
| #53 | (([ALL=SARS-CoV-2] AND ((((((((([ALL=중환자] OR [ALL=중증]) OR [ALL=호흡부전]) OR [ALL=호흡곤란]) OR [ALL=폐렴]) OR [ALL=폐부종]) OR [ALL=폐섬유증]) OR [ALL=기흉]) OR [ALL=종격기종]) OR [ALL=기종격])) AND (([ALL=Intratracheal Intubation] OR [ALL=Endotracheal Intubation]) OR [ALL=early intubation])) |
| #54 | ((([ALL=SARS-CoV-2] OR [ALL=Severe acute respiratory syndrome coronavirus 2]) AND (([ALL=저산소증] OR [ALL=산소 부족]) OR [ALL=산소결핍])) AND (([ALL=Intratracheal Intubation] OR [ALL=Endotracheal Intubation]) OR [ALL=early intubation])) |
| #55 | (([ALL=SARS-CoV-2] AND (((([ALL=고유량] OR [ALL=비침습적 환기]) OR [ALL=비침습적 양압환기]) OR [ALL=간헐적 양압환기]) OR [ALL=간헐적 양압호흡])) AND (([ALL=Intratracheal Intubation] OR [ALL=Endotracheal Intubation]) OR [ALL=early intubation])) |
| #56 | (([ALL=Severe acute respiratory syndrome coronavirus 2] AND ((((((((([ALL=중환자] OR [ALL=중증]) OR [ALL=호흡부전]) OR [ALL=호흡곤란]) OR [ALL=폐렴]) OR [ALL=폐부종]) OR [ALL=폐섬유증]) OR [ALL=기흉]) OR [ALL=종격기종]) OR [ALL=기종격])) AND (([ALL=Intratracheal Intubation] OR [ALL=Endotracheal Intubation]) OR [ALL=early intubation])) |
| #57 | (([ALL=Severe acute respiratory syndrome coronavirus 2] AND (((([ALL=고유량] OR [ALL=비침습적 환기]) OR [ALL=비침습적 양압환기]) OR [ALL=간헐적 양압환기]) OR [ALL=간헐적 양압호흡])) AND (([ALL=Intratracheal Intubation] OR [ALL=Endotracheal Intubation]) OR [ALL=early intubation])) |
| #58 | (((([ALL=COVID-19] OR [ALL=COVID19]) OR [ALL=coronavirus]) AND ((((((((([ALL=중환자] OR [ALL=중증]) OR [ALL=호흡부전]) OR [ALL=호흡곤란]) OR [ALL=폐렴]) OR [ALL=폐부종]) OR [ALL=폐섬유증]) OR [ALL=기흉]) OR [ALL=종격기종]) OR [ALL=기종격])) AND (([ALL=기관 내 삽관] OR [ALL=기관내삽관]) OR [ALL=조기 삽관])) |
| #59 | (((([ALL=COVID-19] OR [ALL=COVID19]) OR [ALL=coronavirus]) AND (([ALL=저산소증] OR [ALL=산소 부족]) OR [ALL=산소결핍])) AND (([ALL=기관 내 삽관] OR [ALL=기관내삽관]) OR [ALL=조기 삽관])) |
| #60 | (((([ALL=COVID-19] OR [ALL=COVID19]) OR [ALL=coronavirus]) AND (((([ALL=고유량] OR [ALL=비침습적 환기]) OR [ALL=비침습적 양압환기]) OR [ALL=간헐적 양압환기]) OR [ALL=간헐적 양압호흡])) AND (([ALL=기관 내 삽관] OR [ALL=기관내삽관]) OR [ALL=조기 삽관])) |
| #61 | (([ALL=SARS-CoV-2] AND ((((((((([ALL=중환자] OR [ALL=중증]) OR [ALL=호흡부전]) OR [ALL=호흡곤란]) OR [ALL=폐렴]) OR [ALL=폐부종]) OR [ALL=폐섬유증]) OR [ALL=기흉]) OR [ALL=종격기종]) OR [ALL=기종격])) AND (([ALL=기관 내 삽관] OR [ALL=기관내삽관]) OR [ALL=조기 삽관])) |
| #62 | (([ALL=SARS-CoV-2] AND (([ALL=저산소증] OR [ALL=산소 부족]) OR [ALL=산소결핍])) AND (([ALL=기관 내 삽관] OR [ALL=기관내삽관]) OR [ALL=조기 삽관])) |
| #63 | (([ALL=SARS-CoV-2] AND (((([ALL=고유량] OR [ALL=비침습적 환기]) OR [ALL=비침습적 양압환기]) OR [ALL=간헐적 양압환기]) OR [ALL=간헐적 양압호흡])) AND (([ALL=기관 내 삽관] OR [ALL=기관내삽관]) OR [ALL=조기 삽관])) |
| #64 | (([ALL=Severe acute respiratory syndrome coronavirus 2] AND ((((((((([ALL=중환자] OR [ALL=중증]) OR [ALL=호흡부전]) OR [ALL=호흡곤란]) OR [ALL=폐렴]) OR [ALL=폐부종]) OR [ALL=폐섬유증]) OR [ALL=기흉]) OR [ALL=종격기종]) OR [ALL=기종격])) AND (([ALL=기관 내 삽관] OR [ALL=기관내삽관]) OR [ALL=조기 삽관])) |
| #65 | (([ALL=Severe acute respiratory syndrome coronavirus 2] AND (([ALL=저산소증] OR [ALL=산소 부족]) OR [ALL=산소결핍])) AND (([ALL=기관 내 삽관] OR [ALL=기관내삽관]) OR [ALL=조기 삽관])) |
| #66 | (([ALL=Severe acute respiratory syndrome coronavirus 2] AND (((([ALL=고유량] OR [ALL=비침습적 환기]) OR [ALL=비침습적 양압환기]) OR [ALL=간헐적 양압환기]) OR [ALL=간헐적 양압호흡])) AND (([ALL=기관 내 삽관] OR [ALL=기관내삽관]) OR [ALL=조기 삽관])) |
| #67 | (((([ALL=코로나-19] OR [ALL=코로나19]) OR [ALL=코로나]) AND (((([ALL=Critical] OR [ALL=Intensive]) OR [ALL=Severe]) OR [ALL=Trauma Centers]) OR [ALL=Critically ill])) AND (([ALL=Intratracheal Intubation] OR [ALL=Endotracheal Intubation]) OR [ALL=early intubation])) |
| #68 | (((([ALL=코로나-19] OR [ALL=코로나19]) OR [ALL=코로나]) AND (((([ALL=Respiratory] OR [ALL=ARDS]) OR [ALL=lung]) OR [ALL=pulmonary]) OR [ALL=fibrosing alveolitis])) AND (([ALL=Intratracheal Intubation] OR [ALL=Endotracheal Intubation]) OR [ALL=early intubation])) |
| #69 | (((([ALL=코로나-19] OR [ALL=코로나19]) OR [ALL=코로나]) AND (((([ALL=pneumothorax] OR [ALL=Mediastinal Emphysema]) OR [ALL=Pneumomediastinum]) OR [ALL=hypoxia]) OR [ALL=hypoxemia])) AND (([ALL=Intratracheal Intubation] OR [ALL=Endotracheal Intubation]) OR [ALL=early intubation])) |
| #70 | (((([ALL=코로나-19] OR [ALL=코로나19]) OR [ALL=코로나]) AND (([ALL=HFNC] OR [ALL=high flow]) OR [ALL=optiflow])) AND (([ALL=Intratracheal Intubation] OR [ALL=Endotracheal Intubation]) OR [ALL=early intubation])) |
| #71 | (((([ALL=코로나-19] OR [ALL=코로나19]) OR [ALL=코로나]) AND ([ALL=NIPPV] OR [ALL=non-invasive positive pressure])) AND (([ALL=Intratracheal Intubation] OR [ALL=Endotracheal Intubation]) OR [ALL=early intubation])) |
| #72 | (((([ALL=코로나-19] OR [ALL=코로나19]) OR [ALL=코로나]) AND ([ALL=BIPAP] OR [ALL=noninvasive positive pressure])) AND (([ALL=Intratracheal Intubation] OR [ALL=Endotracheal Intubation]) OR [ALL=early intubation])) |
| #73 | (((([ALL=코로나-19] OR [ALL=코로나19]) OR [ALL=코로나]) AND ([ALL=non-invasive ventilation] OR [ALL=noninvasive ventilation])) AND (([ALL=Intratracheal Intubation] OR [ALL=Endotracheal Intubation]) OR [ALL=early intubation])) |
| #74 | (((([ALL=코로나-19] OR [ALL=코로나19]) OR [ALL=코로나]) AND [ALL=Intermittent Positive-Pressure Ventilation]) AND (([ALL=Intratracheal Intubation] OR [ALL=Endotracheal Intubation]) OR [ALL=early intubation])) |
| #75 | (((([ALL=코로나-19] OR [ALL=코로나19]) OR [ALL=코로나]) AND (((([ALL=Critical] OR [ALL=Intensive]) OR [ALL=Severe]) OR [ALL=Trauma Centers]) OR [ALL=Critically ill])) AND (([ALL=Intratracheal Intubation] OR [ALL=Endotracheal Intubation]) OR [ALL=early intubation])) |
| #76 | (((([ALL=코로나-19] OR [ALL=코로나19]) OR [ALL=코로나]) AND (((([ALL=Respiratory] OR [ALL=ARDS]) OR [ALL=lung]) OR [ALL=pulmonary]) OR [ALL=fibrosing alveolitis])) AND (([ALL=기관 내 삽관] OR [ALL=기관내삽관]) OR [ALL=조기 삽관])) |
| #77 | (((([ALL=코로나-19] OR [ALL=코로나19]) OR [ALL=코로나]) AND (((([ALL=pneumothorax] OR [ALL=Mediastinal Emphysema]) OR [ALL=Pneumomediastinum]) OR [ALL=hypoxia]) OR [ALL=hypoxemia])) AND (([ALL=기관 내 삽관] OR [ALL=기관내삽관]) OR [ALL=조기 삽관])) |
| #78 | (((([ALL=코로나-19] OR [ALL=코로나19]) OR [ALL=코로나]) AND (([ALL=HFNC] OR [ALL=high flow]) OR [ALL=optiflow])) AND (([ALL=기관 내 삽관] OR [ALL=기관내삽관]) OR [ALL=조기 삽관])) |
| #79 | (((([ALL=코로나-19] OR [ALL=코로나19]) OR [ALL=코로나]) AND ([ALL=NIPPV] OR [ALL=non-invasive positive pressure])) AND (([ALL=기관 내 삽관] OR [ALL=기관내삽관]) OR [ALL=조기 삽관])) |
| #80 | (((([ALL=코로나-19] OR [ALL=코로나19]) OR [ALL=코로나]) AND ([ALL=BIPAP] OR [ALL=noninvasive positive pressure])) AND (([ALL=기관 내 삽관] OR [ALL=기관내삽관]) OR [ALL=조기 삽관])) |
| #81 | (((([ALL=코로나-19] OR [ALL=코로나19]) OR [ALL=코로나]) AND ([ALL=non-invasive ventilation] OR [ALL=noninvasive ventilation])) AND (([ALL=기관 내 삽관] OR [ALL=기관내삽관]) OR [ALL=조기 삽관])) |
| #82 | (((([ALL=코로나-19] OR [ALL=코로나19]) OR [ALL=코로나]) AND [ALL=Intermittent Positive-Pressure Ventilation]) AND (([ALL=기관 내 삽관] OR [ALL=기관내삽관]) OR [ALL=조기 삽관])) |
| #83 | (((([ALL=코로나-19] OR [ALL=코로나19]) OR [ALL=코로나]) AND ((((((((([ALL=중환자] OR [ALL=중증]) OR [ALL=호흡부전]) OR [ALL=호흡곤란]) OR [ALL=폐렴]) OR [ALL=폐부종]) OR [ALL=폐섬유증]) OR [ALL=기흉]) OR [ALL=종격기종]) OR [ALL=기종격])) AND (([ALL=Intratracheal Intubation] OR [ALL=Endotracheal Intubation]) OR [ALL=early intubation])) |
| #84 | (((([ALL=코로나-19] OR [ALL=코로나19]) OR [ALL=코로나]) AND (([ALL=저산소증] OR [ALL=산소 부족]) OR [ALL=산소결핍])) AND (([ALL=Intratracheal Intubation] OR [ALL=Endotracheal Intubation]) OR [ALL=early intubation])) |
| #85 | (((([ALL=코로나-19] OR [ALL=코로나19]) OR [ALL=코로나]) AND (((([ALL=고유량] OR [ALL=비침습적 환기]) OR [ALL=비침습적 양압환기]) OR [ALL=간헐적 양압환기]) OR [ALL=간헐적 양압호흡])) AND (([ALL=Intratracheal Intubation] OR [ALL=Endotracheal Intubation]) OR [ALL=early intubation])) |
| #86 | (((([ALL=코로나-19] OR [ALL=코로나19]) OR [ALL=코로나]) AND ((((((((([ALL=중환자] OR [ALL=중증]) OR [ALL=호흡부전]) OR [ALL=호흡곤란]) OR [ALL=폐렴]) OR [ALL=폐부종]) OR [ALL=폐섬유증]) OR [ALL=기흉]) OR [ALL=종격기종]) OR [ALL=기종격])) AND (([ALL=기관 내 삽관] OR [ALL=기관내삽관]) OR [ALL=조기 삽관])) |
| #87 | (((([ALL=코로나-19] OR [ALL=코로나19]) OR [ALL=코로나]) AND (([ALL=저산소증] OR [ALL=산소 부족]) OR [ALL=산소결핍])) AND (([ALL=기관 내 삽관] OR [ALL=기관내삽관]) OR [ALL=조기 삽관])) |
| #88 | (((([ALL=코로나-19] OR [ALL=코로나19]) OR [ALL=코로나]) AND (((([ALL=고유량] OR [ALL=비침습적 환기]) OR [ALL=비침습적 양압환기]) OR [ALL=간헐적 양압환기]) OR [ALL=간헐적 양압호흡])) AND (([ALL=기관 내 삽관] OR [ALL=기관내삽관]) OR [ALL=조기 삽관])) |
| #89 | OR/1-88 |
