# Supplementary material for: Early intubation and clinical outcomes in patients with severe COVID-19: a systematic review and meta-analysis
Source: Eur J Med Res. 2022 Nov 3;27:226. doi: 10.1186/s40001-022-00841-6 (PMC9631590; doi:10.1186/s40001-022-00841-6)
Supplement: Supplementary file 3 — Additional file 3. List of excluded studies after full-text screening [file 40001_2022_841_MOESM3_ESM.docx]

**Additional file 3. List of excluded studies after full-text screening**

| **No** | **Excluded studies** | **Reason for exclusion** |
| --- | --- | --- |
| 1 | Angulo MAA: Transmission of COVID-19 among the health personnel of the Hospital Victor lazarte echegaray in Trujillo. [Spanish]. Horizonte Medico 2021, 21 (1) (no pagination)(e1371). | Other than English or Korean |
| 2 | Bayrak V, Senturk Durukan N, Demirer Aydemir F, Ergan B, Gezer NS, Eren Kutsoylu OO, Gokmen AN, Savran Y: Risk factors associated with mortality in intensive care COVID-19 patients: the importance of chest CT score and intubation timing as risk factors. Turkish Journal of Medical Sciences 2021, 51(4):1665-1674. | Study without early intubation in the intervention group |
| 3 | Boscolo A, Pasin L, Sella N, Pretto C, Tocco M, Tamburini E, Rosi P, Polati E, Donadello K, Gottin L et al: Outcomes of COVID-19 patients intubated after failure of non-invasive ventilation: a multicenter observational study. Scientific Reports 2021, 11(1):17730. | Study without early intubation in the intervention group |
| 4 | Cabrini L, Ghislanzoni L, Severgnini P, Landoni G, Baiardo Redaelli M, Franchi F, Romagnoli S: Early versus late tracheal intubation in COVID-19 patients: a pro-con debate also considering heart-lung interactions. Minerva cardioangiologica 2020, 15. | Study other than randomized controlled trials or observational studies |
| 5 | Cai SJ, Wu LL, Chen DF, Li YX, Liu YJ, Fan YQ, Du SH, Huang H, Liu N, Cheng LL et al: [Analysis of bronchoscope-guided tracheal intubation in 12 cases with coronavirus disease 2019 under the personal protective equipment with positive pressure protective hood]. Zhonghua Jie He He Hu Xi Za Zhi 2020, 43(4):332-334. | Other than English or Korean |
| 6 | Cai XF, Sun JM, Dong ZQ, Li WB: Invasive fungal infections in the pediatric intensive care unit: A clinical analysis of 38 cases. [Chinese]. Chinese Journal of Contemporary Pediatrics 2013, 15(8):644-648. | Other than English or Korean |
| 7 | Carrillo Hernandez-Rubio J, Sanchez-Carpintero Abad M, Yordi Leon A, Doblare Higuera G, Garcia Rodriguez L, Garcia Torrejon C, Mayor Cacho A, Jimenez Rodriguez A, Garcia-Salmones Martin M: Outcomes of an intermediate respiratory care unit in the COVID-19 pandemic. PLoS One 2020, 15(12):e0243968. | Study without early intubation in the intervention group |
| 8 | Ctri: Comparison of two different types of simple non-invasive oxygen therapy devices in coronavirus lung infection. http://wwwwhoint/trialsearch/Trial2aspx?TrialID=CTRI/2020/07/026835 2020. | Ongoing study |
| 9 | Daniel P, Mecklenburg M, Massiah C, Joseph MA, Wilson C, Parmar P, Rosengarten S, Maini R, Kim J, Oomen A et al: Non-invasive positive pressure ventilation versus endotracheal intubation in treatment of COVID-19 patients requiring ventilatory support. Am J Emerg Med 2021, 43:103-108. | Study without early intubation in the intervention group |
| 10 | Diaz De Teran T, Gonzales Martinez M, Banfi P, Garuti G, Ferraioli G, Russo G, Casu F, Vivarelli M, Bonfiglio M, Perazzo A et al: Management of patients with severe acute respiratory failure due to SARS-CoV-2 pneumonia with noninvasive ventilatory support outside Intensive Care Unit. Minerva Medica 2021, 112(3):329-337. | Study without early intubation in the intervention group |
| 11 | Estenssoro E, Loudet CI, Rios FG, Kanoore Edul VS, Plotnikow G, Andrian M, Romero I, Piezny D, Bezzi M, Mandich V et al: Clinical characteristics and outcomes of invasively ventilated patients with COVID-19 in Argentina (SATICOVID): a prospective, multicentre cohort study. The Lancet Respiratory Medicine 2021, 9(9):989-998. | Study without early intubation in the intervention group |
| 12 | Ferreyro BL, Angriman F, Hernandez-Sanz M, Arruti E, Torres A, Villar J, Brochard L, Ferrando C, Mellado-Artigas R, Vendrell M et al: High-flow nasal oxygen in patients with COVID-19-associated acute respiratory failure. Critical Care 2021, 25 (1) (no pagination)(58). | Duplication |
| 13 | Garduno-Lopez AL, Guido-Guerra RE, Guizar-Rangel MT, Acosta-Nava VM, Dominguez-Cherit G, Alvarez-Bobadilla G: Perioperative management of the patient with COVID-19. [Spanish]. Revista Mexicana de Anestesiologia 2020, 43(2):109-120. | Other than English or Korean |
| 14 | Gershengorn HB, Hu Y, Chen JT, Hsieh SJ, Dong J, Gong MN, Chan CW: The Impact of High-Flow Nasal Cannula Use on Patient Mortality and the Availability of Mechanical Ventilators in COVID-19. Ann Am Thorac Soc 2021, 18(4):623-631. | Study other than randomized controlled trials or observational studies |
| 15 | Gervasio CF, Averono G, Robiolio L, Bertoletti M, Colageo U, De Col L, Bertone F: Tracheal Stenosis After Tracheostomy for Mechanical Ventilation in COVID-19 Pneumonia - A Report of 2 Cases from Northern Italy. Am J Case Rep 2020, 21:e926731. | Study other than randomized controlled trials or observational studies |
| 16 | Guevara-Valerio H, Mari-Zapata DD: Considerations in the management of the airway in the patient with COVID-19. [Spanish]. Medicina Interna de Mexico 2020, 36(3):318-322. | Other than English or Korean |
| 17 | Herbstreit F, Welsner M, Taube C, Ernst EC, Stoppler T, Brenner T, Schmidt K: ECMO as a bridging to recovery in a 19-year-old patient with e-cigarette / vaping-product associated lung injury (EVALI). [German]. Anasthesiologie und Intensivmedizin 2020, 61(12):605-608. | Other than English or Korean |
| 18 | Istvan L, Csilla M, Gyorgy K, Tamas V, Akos F, Mariann B, Marianna J, Bela F: Airway management of coronavirus-infected patients. [Hungarian]. Orvosi Hetilap 2020, 161(17):696-703. | Other than English or Korean |
| 19 | Karagiannidis C, Hentschker C, Westhoff M, Weber-Carstens S, Janssens U, Kluge S, Pfeifer M, Spies C, Welte T, Rossaint R et al: Observational study of changes in utilization and outcomes in mechanical ventilation in COVID-19. PLoS ONE [Electronic Resource] 2022, 17(1):e0262315. | Study without early intubation in the intervention group |
| 20 | Karasu D, Eminoglu S, Ozgunay SE, Gamli M: The knowledge levels and attitudes of anesthesia technicians about intubation of patients with positive or suspected coronavirus (COVID-19). [Turkish]. Anestezi Dergisi 2021, 29(1):255-260. | Other than English or Korean |
| 21 | Lei ZM, Zhang M: Study of root canal filling in dogs by using compound coral paste. [Chinese]. Journal of Clinical Rehabilitative Tissue Engineering Research 2008, 12(10):1983-1986. | Other than English or Korean |
| 22 | Leister N, Yucetepe S, Ulrichs C, Hannes T, Trieschmann U: SARS-CoV-2 positive child-What to do if inhalation induction of anesthesia is unavoidable?. [German]. Anaesthesist 2021. | Other than English or Korean |
| 23 | Liping J, Changfeng W, Yujiao Z, Yuanyuan Y, Zhaoxia J: Clinical characteristics of coronavirus disease 2019 patients complicated with pneumothorax: Analysis of 7 cases. [Chinese]. Zhonghua Wei Zhong Bing Ji Jiu Yi Xue 2020, 32(10):1174-1177. | Other than English or Korean |
| 24 | Liu Z, Zhao HY, Zuo MZ: Tracheal intubation in patients with coronavirus disease 2019 (COVID-19): a cross-sectional survey in China. Chinese Medical Journal 2021, 134(17):2110-2112. | Study without early intubation in the intervention group |
| 25 | Luna-Castaneda AC, Sanchez-Gonzalez A, Buenrostro-Gaitan A, Bernardino-Gonzalez AK, Gutierrez-Bautista AK, Flores-Martinez A, Pichardo-Estrada A, Martinez-Martinez BE, Aguilar-Viveros B, Galeana-Miramontes C et al: Biosecurity and airway management in the pediatric patient with SARS-CoV-2 infection. [Spanish]. Neumologia y Cirugia de Torax(Mexico) 2020, 79(4):265-277. | Other than English or Korean |
| 26 | Mellado-Artigas R, Mujica LE, Ruiz ML, Ferreyro BL, Angriman F, Arruti E, Torres A, Barbeta E, Villar J, Ferrando C et al: Predictors of failure with high-flow nasal oxygen therapy in COVID-19 patients with acute respiratory failure: a multicenter observational study. Journal of Intensive Care 2021, 9 (1) (no pagination)(23). | Study without early intubation in the intervention group |
| 27 | Mullington CJ, Shetty P, Dalton J: Intubation of COVID patients: always a risky business? Anaesthesia 2021, 76 Suppl 3:3-4. | Study without early intubation in the intervention group |
| 28 | Nct: Hydroxychloroquine in SARS-CoV-2 (COVID-19) Pneumonia Trial. https://clinicaltrialsgov/show/NCT04382625 2020. | Ongoing study |
| 29 | Nct: Early Versus Delayed Intubation of Patients With COVID-19. https://clinicaltrialsgov/show/NCT04632043 2020. | Ongoing study |
| 30 | Oates CP, Goldman SA, Giustino G, Goldman ME: Trimming the need for invasive ventilation: pragmatic critical care during the COVID-19 pandemic. BMJ Case Rep 2020, 13(9). | Study without early intubation in the intervention group |
| 31 | Pfeifer M, Ewig S, Voshaar T, Randerath W, Bauer T, Geiseler J, Dellweg D, Westhoff M, Windisch W, Schonhofer B et al: Position Paper for the State of the Art Application of Respiratory Support in Patients with COVID-19: German Respiratory Society. [German]. Pneumologie 2020, 74(6):337-357. | Other than English or Korean |
| 32 | Pfeifer M, Ewig S, Voshaar T, Randerath W, Bauer T, Geiseler J, Dellweg D, Westhoff M, Windisch W, Schönhofer B et al: [Position Paper for the State of the Art Application of Respiratory Support in Patients with COVID-19 - German Respiratory Society]. Pneumologie 2020, 74(6):337-357. | Duplication |
| 33 | Sheikh S, Baig MA: Optimising Ventilator Use during the COVID-19 Pandemic. J Coll Physicians Surg Pak 2020, 30(6):46-47. | Study without early intubation in the intervention group |
| 34 | Sherly Deborah G, Archana R, Narayanam H: Using the mask - do's and don'ts in the COVID-19 scenario. International Journal of Research in Pharmaceutical Sciences 2020, 11(Special Issue 1):901-906. | Study other than randomized controlled trials or observational studies |
| 35 | Siempos, II, Xourgia E, Ntaidou TK, Zervakis D, Magira EE, Kotanidou A, Routsi C, Zakynthinos SG: Effect of Early vs. Delayed or No Intubation on Clinical Outcomes of Patients With COVID-19: An Observational Study. Front Med (Lausanne) 2020, 7:614152. | Duplication |
| 36 | Stais P, Salloum O, Kühle D, Fisteag S, Kambartel K, Veelken D, Ewig S, Voshaar T: [48-Year-Old Triathlete with Severe COVID-19 Pneumonia: Successful and Safe Treatment with Oxygen and CPAP]. Pneumologie 2020, 74(7):417-422. | Other than English or Korean |
| 37 | Vera M, Kattan E, Born P, Rivas E, Amthauer M, Nesvadba A, Lara B, Rao I, Espindola E, Rojas L et al: Intubation timing as determinant of outcome in patients with acute respiratory distress syndrome by SARS-CoV-2 infection. Journal of Critical Care 2021, 65:164-169. | Duplication |
| 38 | Vianello A, Arcaro G, Molena B, Turato C, Sukthi A, Guarnieri G, Lugato F, Senna G, Navalesi P: High-flow nasal cannula oxygen therapy to treat patients with hypoxemic acute respiratory failure consequent to SARS-CoV-2 infection. Thorax 2020, 75(11):998-1000. | Study without early intubation in the intervention group |
| 39 | Wang J, Lu F, Zhou M, Qi Z, Chen Z: Tracheal intubation in patients with severe and critical COVID-19: analysis of 18 cases. [Chinese]. Nan Fang Yi Ke Da Xue Xue Bao 2020, 40(3):337-341. | Other than English or Korean |
| 40 | Wang J, Lu F, Zhou M, Qi Z, Chen Z: [Tracheal intubation in patients with severe and critical COVID-19: analysis of 18 cases]. Nan Fang Yi Ke Da Xue Xue Bao 2020, 40(3):337-341. | Duplication |
| 41 | Xiao-Ling Z, Rui-Yong C, Xiang-Qun N, Xiao-Lan T, Yi L, Yan-Chao T: Hyperbaric oxygen therapy in an elderly critical coronavirus disease 2019 patient with endotracheal intubation: clinical effect analysis. [Chinese]. Academic Journal of Second Military Medical University 2020, 41(6):621-627. | Other than English or Korean |
| 42 | Zirpe KG, Tiwari AM, Gurav SK, Deshmukh AM, Suryawanshi PB, Wankhede PP, Kapse US, Bhoyar AP, Khan AZ, Malhotra RV et al: Timing of invasive mechanical ventilation and mortality among patients with severe COVID-19-associated acute respiratory distress syndrome (CARDS). Indian Journal of Critical Care Medicine 2021, 25(5):493-498. | Duplication |
