# Supplementary material for: Early intubation and clinical outcomes in patients with severe COVID-19: a systematic review and meta-analysis
Source: Eur J Med Res. 2022 Nov 3;27:226. doi: 10.1186/s40001-022-00841-6 (PMC9631590; doi:10.1186/s40001-022-00841-6)
Supplement: Supplementary file 4 — Additional file 4. Forest plots. Figure S1. Subgroup analysis of length of stay in ICU by definition of early intubation as < 24 h or < 48 h from index time. Figure S2. Subgroup analysis of length of stay in ICU by index time in studies defining early intubation as < 24 h (a) or < 48 h (b). Figure S3. Subgroup analysis of duration of mechanical ventilation by definition of early intubation as < 24 h or < 48 h from index time. Figure S4. Subgroup analysis of duration of mechanical ventilation by index time in studies defining early intubation as < 24 h (a) or < 48 h (b). Figure S5. Hospital length of stay. Figure S6. Subgroup analysis of hospital length of stay by definition of early intubation as < 24 h or < 48 h from index time. Figure S7. Ventilator-free days. Figure S8. ICU mortality. Figure S9. 28-day mortality [file 40001_2022_841_MOESM4_ESM.docx]

**Additional file 4.**

**Figure S1. Subgroup analysis of length of stay in ICU by definition of early intubation as < 24 hours or < 48 hours from index time**

**
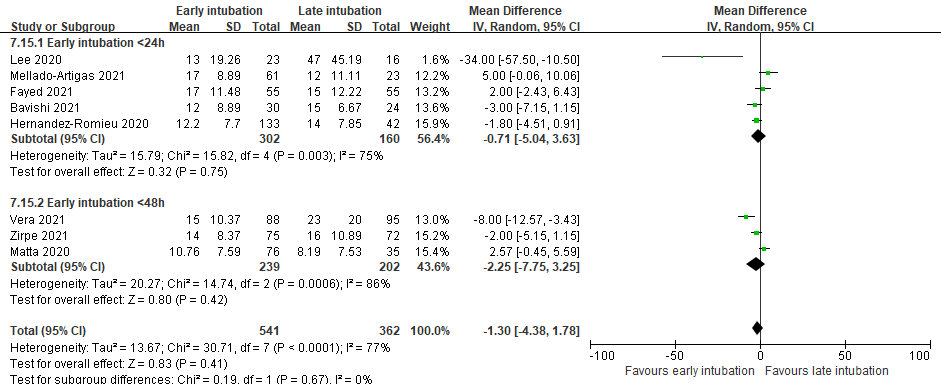
**

**Figure S2. Subgroup analysis of length of stay in ICU by index time in studies defining early intubation as < 24 hours (a) or <48 hours (b)**

**(a) Subgroup analysis of length of stay in ICU by index time in studies defining early intubation as < 24 hours**

**
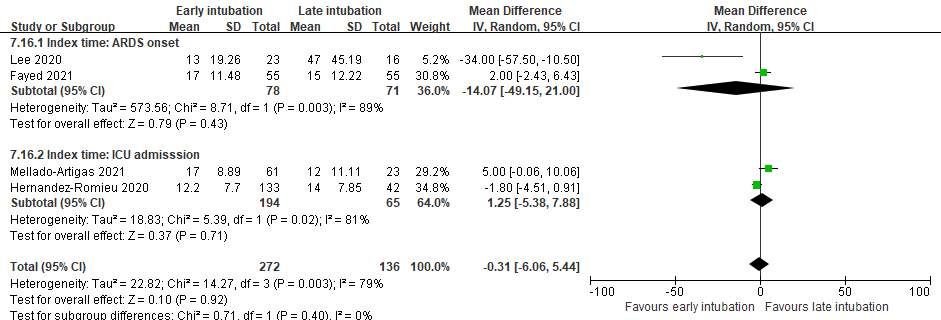
**

**(b) Subgroup analysis of length of stay in ICU by index time in studies defining early intubation as <48 hours**

**
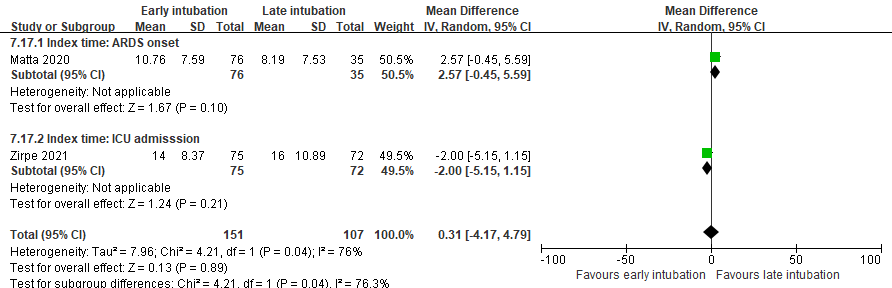
**

**Figure S3. Subgroup analysis of duration of mechanical ventilation by definition of early intubation as < 24 hours or < 48 hours from index time**

**
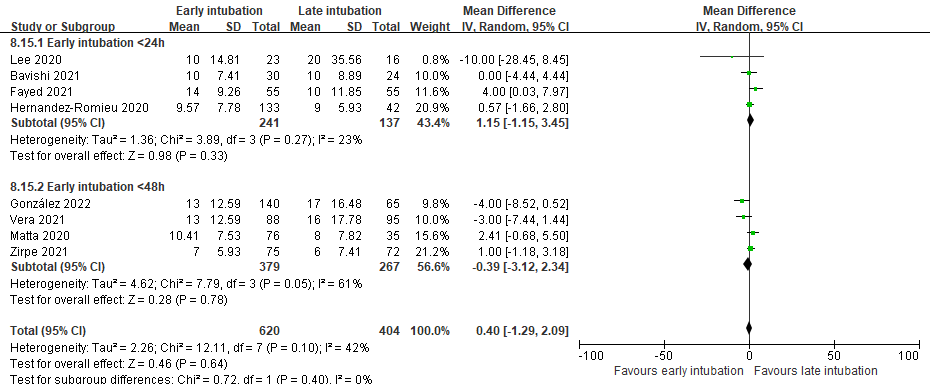
**

**Figure S4. Subgroup analysis of duration of mechanical ventilation by index time in studies defining early intubation as < 24 hours (a) or <48 hours (b)**

**(a) Subgroup analysis of duration of mechanical ventilation by index time in studies defining early intubation as < 24 hours**

**
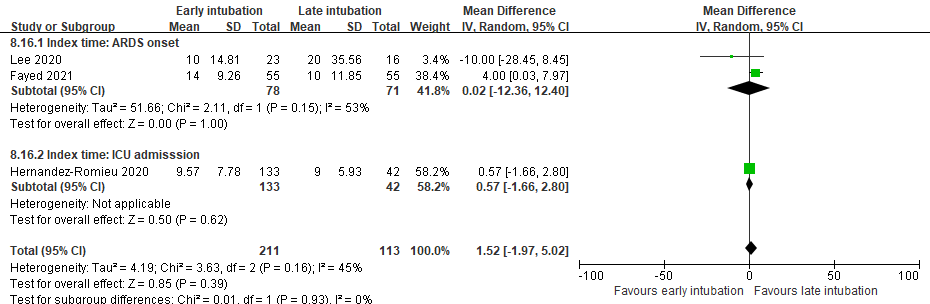
**

**(b) Subgroup analysis of duration of mechanical ventilation by index time in studies defining early intubation as <48 hours**

**
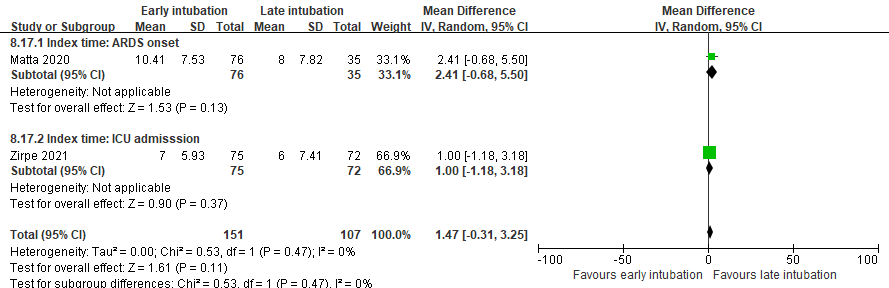
**

**Figure S5. Hospital length of stay**

**
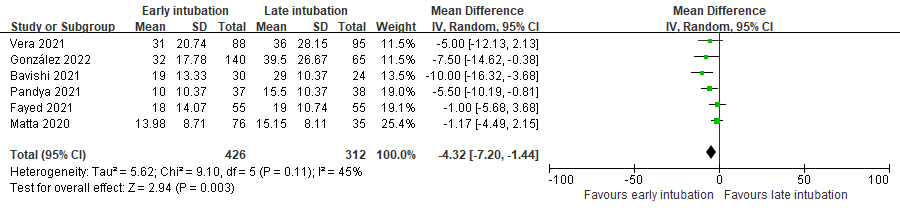
**

**Figure S6. Subgroup analysis of hospital length of stay by definition of early intubation as < 24 hours or < 48 hours from index time**

**
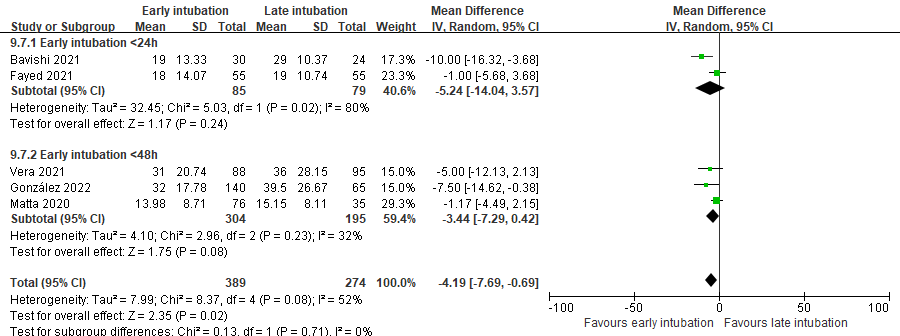
**

**Figure S7. Ventilator-free days**

**
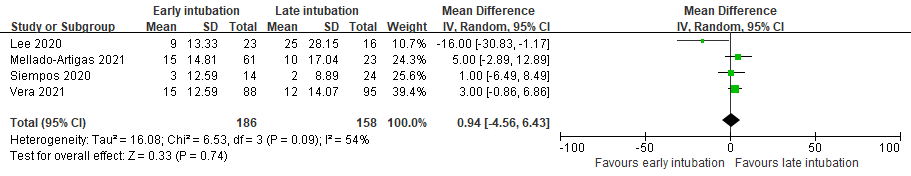
**

**Figure S8. ICU mortality**

**
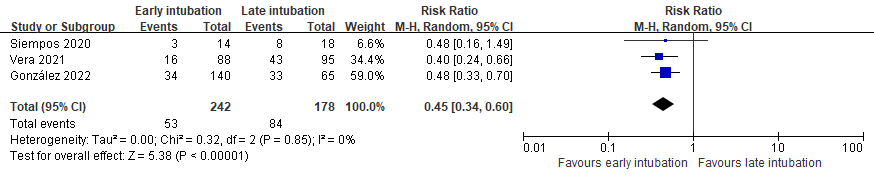
**

**Figure S9. 28-day mortality**

**
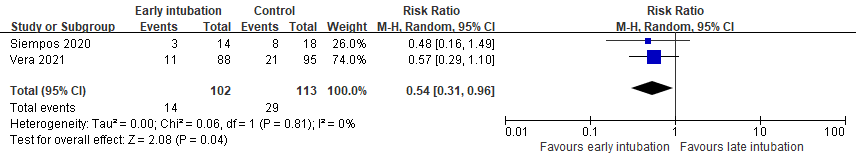
**
